# Enhanced Microglial Engulfment of Dopaminergic Synapses Induces Parkinson's Disease‐Related Executive Dysfunction in an Acute LPC Infusion Targeting the mPFC

**DOI:** 10.1111/acel.70003

**Published:** 2025-02-15

**Authors:** Yehao Liu, Rui Chen, Chunyan Mu, Junjie Diao, Yurong Guo, Xiaoyu Yao, Shijie Shi, Mengying Wang, Zhi Zhang, Xiaoling Qin, Chuanxi Tang

**Affiliations:** ^1^ Department of Neurobiology, Xuzhou Key Laboratory of Neurobiology Xuzhou Medical University Xuzhou Jiangsu China; ^2^ School of Basic Medical Sciences Anhui Medical University Hefei China; ^3^ Department of Neurology The Second People's Hospital of Huai'an and the Affiliated Huai'an Hospital of Xuzhou Medical University Jiangsu China; ^4^ Department of Epidemiology and Biostatistics, School of Public Health Peking University Health Science Center Beijing China; ^5^ Department of Neurology Shanghai Xuhui Central Hospital, Zhongshan‐Xuhui Hospital, Fudan University Shanghai China

**Keywords:** 5‐CSRT, C1q, dopaminergic synapses, executive dysfunction, Lysophosphatidylcholine, medial prefrontal cortex, Parkinson's disease, phosphatidylserine

## Abstract

The dysfunction of the dopaminergic projection from the ventral tegmental area (VTA) to the medial prefrontal cortex (mPFC) is believed to play a key role in the pathophysiology of Parkinson's disease (PD) accompanied by executive dysfunction (EDF). In this study, we identified an abnormal increase in lysophosphatidylcholine (LPC) levels in PD patients, which closely correlates with the severity of cognitive impairment. LPC disrupts the miR‐2885/TDP‐43 signaling pathway in microglia, driving dopaminergic presynaptic engulfment. In LPC‐exposed mice, microglial activation via miR‐2885/TDP‐43/p65 signaling led to inflammatory cytokine and complement release, marking dopaminergic synapses for phagocytosis with a “PS/C1q” signal. Following the inhibition of LPC‐induced microglial activation through chemogenetic methods, we observed a significant reduction in the phagocytosis of dopaminergic synapses, resulting in improved executive function. The miR‐2885 disrupted LPC‐induced dopaminergic phagocytosis and alleviated EDF. Furthermore, the accumulation of excessive TDP‐43 due to the loss of miR‐2885 promoted the engulfment of dopaminergic synapses by facilitating the entry of p65 into the nucleus. Inhibiting TDP‐43 levels effectively mitigated LPC‐induced EDF. Additionally, supplementing dopamine receptor agonists enhanced the excitability of regional glutamatergic neurons, leading to improved executive function. In summary, LPC exposure in the mPFC impairs microglial regulation, leading to dopaminergic synaptic loss and underactivity of glutamatergic neurons. These changes drive the development of executive dysfunction in PD.

## Graphical abstract

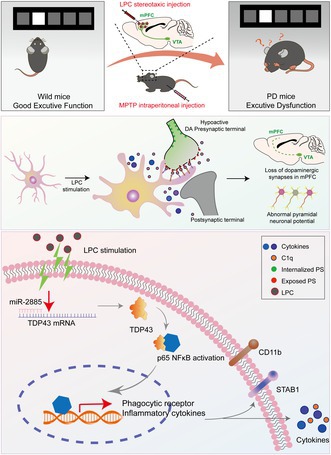

## 1 Introduction

There is substantial evidence supporting the crucial role of the prefrontal cortex in regulating executive functions (Funahashi and Andreau 2013). During tasks involving executive cognition, dopaminergic terminals originating from the ventral tegmental area (VTA) can release dopamine, thus modulating the activity of medial prefrontal cortex (mPFC) neurons (Zhou et al. 2024). Regarding performance, the loss of this dopamine circuit leads to impairments in cognitive flexibility, the ability to adjust behavior for goal‐oriented and intelligent actions, attention allocation, and other aspects related to executive function disorders (Lohani et al. 2019; Tang et al. 2023; Zhou et al. 2024). Clinical drug interventions in PD confirm that dopamine medications have a modest capacity to improve cognitive abilities associated with this pathway in PD patients, particularly concerning working memory (Lewis et al. 2003; Zhou et al. 2024). Hence, it is evident that the integrity and functionality of the VTA‐mPFC dopamine circuit are crucial for normal executive function in PD. However, the specific lipid metabolites involved in dopaminergic terminal degeneration in the mPFC, and the detailed mechanism, remain uncertain.

In previous research, we identified elevated serum levels of lysophosphatidylcholine (LPC) in PD patients with cognitive impairment compared to those without, as part of a comprehensive serum metabolic analysis (Zhang et al. 2021). Similar evidence indicates substantial changes in specific serum lipids, including the LPC family, which may serve as valuable markers for diagnosing and tracking the progression of PD (Lopez de Frutos et al. 2022; Dahabiyeh et al. 2023). Furthermore, LPC has been implicated in mediating alpha‐synuclein (α‐syn)‐induced clustering of synaptic vesicles, linking it to synaptic dysfunction in neurodegenerative processes (Lai et al. 2023). Further proteomic characterization combined with Ingenuity pathway analysis indicated that the regulatory network associated with PD cognitive impairment primarily involves the immune system and inflammatory pathways (Zhang et al. 2021). Given LPC's role as an endogenous inflammatory phospholipid (Jeong et al. 2017), it has been proposed to contribute to microglial activation, which is a key factor in PD pathogenesis (Crowell Jr. et al. 1993; Cui et al. 2014). Building on these findings, we hypothesize that LPC might play a role in the degeneration of dopaminergic terminals in the medial prefrontal cortex (mPFC) via inflammatory mechanisms. Microglia demonstrate remarkable regional specificity and adaptability to their extracellular environment (De Schepper, Crowley, and Hong 2021). Amoeboid microglia, considered ‘activated microglia’, play roles in inflammation, synapse pruning, and neuronal connectivity (Ouchi et al. 2005). Recent research has highlighted the involvement of inflammation‐related microglia–neuron interactions in the anterior cingulate cortex (ACC) in depression, emphasizing the importance of microglial engulfment around ACC glutamatergic neuronal (ACC^Glu^) spines (Cao et al. 2021). This raises the question of whether similar processes are involved in the loss of dopaminergic synapses in the mPFC in PD. Therefore, we investigated here whether abnormal increased LPC can induce the inflammatory processes and phagocytosis of dopaminergic synapses in the mPFC and premature degeneration of executive function in PD. Previous studies have demonstrated the involvement of neuroinflammation and microglial activation in the progression of neurodegenerative disorders, including PD, yet the specific role of LPC in this context remains underexplored. In this study, we investigated the impact of LPC on cognitive function and pathological outcomes, including microglial activation and the phagocytosis of dopaminergic terminals. To further explore the critical role of dopaminergic synaptic loss in mediating EDF, we demonstrated that LPC exacerbates the degeneration of DA terminals and induces EDF through experiments such as dopamine replacement rescue. Additionally, we mitigated EDF by using chemogenetic techniques to block LPC‐induced microglial activation. Furthermore, we examined the transcriptional phenotype of microglial activation following LPC exposure, identifying key miRNAs and conducting experimental validation. This study not only deepens our understanding of the relationship between abnormally elevated LPC and dopaminergic terminal degeneration in the prefrontal cortex but also provides new insights into the complexity of PD‐related executive dysfunction.

## 2 Experimental Model and Subject Details

See File S2.

## 3 Results

### 3.1 Plasma LPC Associates With Cognitive Performance in Parkinson's Disease, Especially in Terms of Executive Function

**FIGURE 1:**
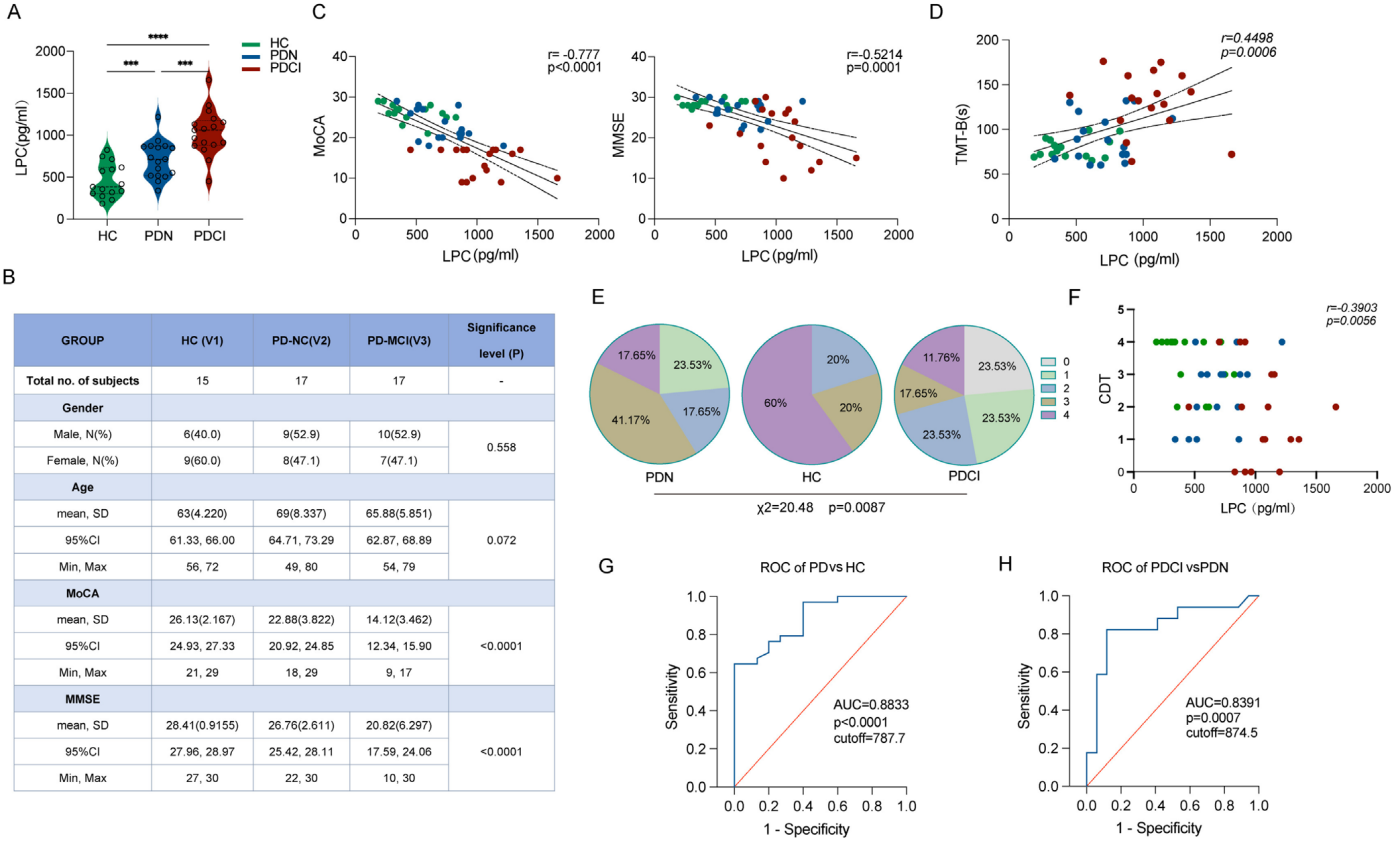
Serum LPC unveiling links to Parkinson's disease, cognitive impairment, and diagnostic precision analysis. (A) Violin plots depicting variations in serum LPC levels (pg/mL) across the Healthy Controls (HC), Parkinson's disease without Cognitive Impairment (PD‐NC), and Parkinson's Disease with Cognitive Impairment (PD‐CI) groups. (B) Comprehensive presentation of demographic information and statistical analysis, including gender, age, Montreal Cognitive Assessment (MoCA) scores, and Mini‐Mental State Examination (MMSE) scores for the HC, PD‐NC, and PD‐CI groups.(C) Exploration of the correlation between serum LPC levels (pg/mL) and cognitive performance assessed by MoCA and MMSE scores. (D) Investigation into the correlation between serum LPC levels (pg/mL) and Trail Making Test Part B (TMT‐B) results, a measure of executive function. (E) Analysis of the composition ratio of Clock Drawing Test (CDT) results (0~5) among the HC, PD‐NC, and PD‐CI groups. (F) Correlation analysis examining the relationship between serum LPC levels (pg/mL) and performance on the Clock Drawing Test (CDT). (G) Receiver Operating Characteristic (ROC) curve illustrating the diagnostic potential of serum LPC levels in distinguishing Parkinson's Disease (PD) from Healthy Controls (HC). (H) ROC curve highlighting the diagnostic efficacy of serum LPC levels in discriminating between PD with Mild Cognitive Impairment (PD‐MCI) and PD without Cognitive Impairment (PD‐NC). Statistical significance denoted as ***p < 0.001 and ****p < 0.0001.

In this study, we enrolled a total of 49 patients to conduct a comprehensive analysis of circulating LPC in PD. Our investigation involved the examination of serum LPC levels in both healthy controls and PD patients, aiming to validate its association with cognitive function. Preliminary findings revealed a significant elevation in LPC levels among PD patients, with a more pronounced increase observed in those with cognitive impairment (PD‐CI) (Figure 1A). To provide a demographic overview, we presented baseline characteristics, including gender, age, and cognitive scores (MoCA, MMSE) in Figure 1B. Notably, there were no significant differences in age (*p* = 0.072) and gender (*p* = 0.558). Correlation analyses unveiled an inverse relationship between serum LPC and cognitive scores, with significant negative correlations observed for MoCA (*r* = −0.777, *p* < 0.001) and MMSE (*r* = −0.5214, *p* = 0.0001) (Figure 1C).

Given that executive dysfunction is a prominent cognitive impairment in PD patients, we further explored the connection between serum LPC concentrations and executive function assessed through TMT‐B and CDT. Significant differences in CDT evaluation results were noted among the three groups (Figure 1E, *p* = 0.0087). Serum LPC levels demonstrated a significant negative correlation with CDT scores (Figure 1F, *r* = −0.3903, *p* = 0.0056) and a positive correlation with TMT‐B (Figure 1D, *r* = 0.4498, *p* = 0.0006). In terms of diagnostic accuracy, ROC analysis revealed that a serum LPC cutoff value of 787.7 pg/mL exhibited a sensitivity of 64.71% and specificity of 93.3% in distinguishing PD from healthy controls, with an area under the curve of 0.8833 (Figure 1G). Similarly, a serum LPC cutoff value of 874.5 pg/mL demonstrated a sensitivity of 82.35% and specificity of 88.24% in distinguishing PD patients with or without cognitive impairment, yielding an area under the curve of 0.8391 (Figure 1G). These results underscore the potential of serum LPC levels as a valuable biomarker for discriminating PD and assessing cognitive impairment in this patient population.

### 3.2 The mPFC Inflammation Induced by LPC Primes Microglial Activation That Affects PD‐Related Executive Function Performance

Given the potential association of LPC with executive dysfunction in PD patients, our study aimed to investigate this hypothesis using a mouse model induced by intraperitoneal injection of MPTP. Studies have demonstrated that MPP+ can enhance the activity of phosphatidylethanolamine‐N‐methyltransferase (PNMT), resulting in elevated levels of LPC (Lee and Charlton 2001). In our preliminary experiments, we assessed PNMT levels in the substantia nigra and prefrontal cortex (PFC) of Parkinson's disease (PD) mice. The results from quantitative PCR (qPCR) indicated a significant increase in PNMT expression (Figure S2A). While the correlation was identified in peripheral samples, we chose to focus our experiments on the medial prefrontal cortex (mPFC) due to its critical role in executive function and its susceptibility to dopaminergic degeneration in PD. In PD brain organoids (GSE208781), the levels of *LCAT, PLA2g6, PLA2g7*, and *PLATT3*, which promote the production of LPC, were significantly elevated. Conversely, the expression of genes involved in LPC degradation, namely *LPCAT2* and *LPCAT4*, was found to be reduced. In the mPFC and substantia nigra of PD mice, the expression of *Lcat, Pla2g2a*, and *Pla2g6* was significantly increased, while the enzymes responsible for LPC degradation (*Lpcat, Enpp2, Lypla*) exhibited no significant changes. Additionally, ELISA results indicated elevated LPC levels in the brain tissues of PD mice (Figure S2B,C).

**FIGURE 2:**
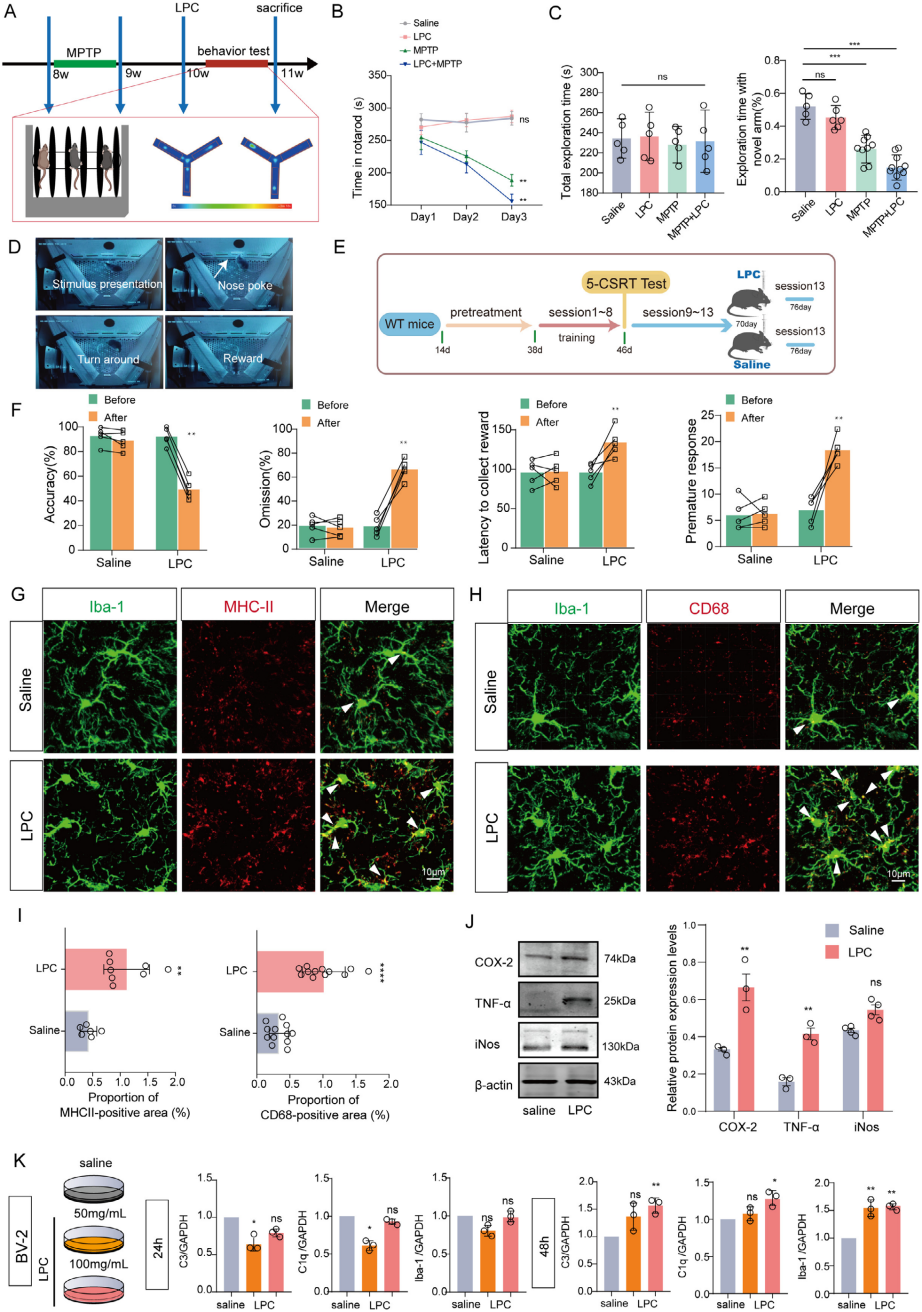
Introduction of exogenous LPC into the mPFC initiates microglial activation, and subsequent executive dysfunction. (A) Flowchart depicting the sequence of drug injection and behavioral phenotype testing in mice. (B) Comparative analysis of the average time on the rotarod on the 3rd day (*n* = 6). (C) Evaluation of total exploration time (*n* = 5, demonstrating no significant difference between groups) and the percentage of exploration in the new arm in the Y‐maze. (D) Schematic representation of the 5‐CSRT touchscreen behavioral experiment, where mice received sugar water rewards upon correct touchscreen responses. (E) Timeline illustration of the 5‐CSRT experiment. (F) Assessment of executive function before and after drug intervention in mice from the Saline and LPC groups, including accuracy, loss rate, reward delay, and immature response (*n* = 5). (G) Co‐scale plot illustrating immunofluorescence of the inflammatory marker MHCII with Iba1. (H) Co‐scale plot illustrating immunofluorescence of the lysosomal marker CD68 with Iba1. (I) Statistics of MHCII and CD68 positive area. (J) Protein immunoblot bands for the inflammatory cytokines COX‐2 and TNF‐α, along with expression level statistics for iNOS. (K) qPCR results statistics for C3, C1q, and Iba1 after 24 h of LPC intervention in BV2 cells in vitro. Additional statistics for LPC intervention at 48 h, including C3, C1q, and Iba1. In all statistical analyses, each data point corresponds to one mouse or cell sample, and the data are presented as mean ± SEM. *p < 0.05, **p < 0.01, ****p < 0.0001, while “ns” denotes no significant difference.

At the 10‐week mark, we administered exogenous LPC to the mice, thereby augmenting LPC levels in the mPFC, and subsequently evaluated their cognitive and motor function performance (Figure 2A). To mitigate any confounding effects on behavioral performance stemming from impaired motor capacity post‐drug injection, we conducted a transfer experiment involving four groups of mice (Saline, LPC, MPTP, and MPTP + LPC). Importantly, motor function impairment was observed solely in the MPTP‐induced Parkinson's mouse model groups, with LPC injection alone exhibiting no detrimental impact on exercise capacity (Figure 2B, *n* = 6, Saline vs. LPC, *p* = 0.9980; Saline vs. MPTP, *p* = 0.0003; Saline vs. MPTP+LPC, *p* = 0.0002). MPTP‐treated C57BL/6 mice exhibited motor deficits, as evidenced by their performance in the rotarod, open‐field, and gait analysis tests, further confirming the findings of our study (Figure S3A–C) (Video can seen in Videos 1, 2, 3, 4, 5) In the Y‐maze experiment, the LPC + MPTP group displayed a significant reduction (approximately 20%) in exploration time in the unknown arm. Intriguingly, no significant difference was noted in the LPC group (Figure 2C, *n* = 6, Saline vs. LPC, *p* = 0.7170; Saline vs. MPTP, *p* = 0.0002; Saline vs. MPTP+LPC, *p* < 0.001). However, a more nuanced evaluation of executive ability through the 5CSRT touchscreen behavior revealed significant differences in the LPC group compared to the Saline group. Evaluation indicators included accuracy (80% Saline, 50% LPC) (*n* = 5, *t* = 9.713, *p* = 0.0006), omission (20% Saline, 45% LPC) (*n* = 5, *t* = 9.893, *p* = 0.0006), latency to collect reward (50 Saline, 75 LPC) (*n* = 5, *t* = 3.927, *p* = 0.0171), and premature response (5 Saline, 15 LPC) (*n* = 5, *t* = 5.530, *p* = 0.0052). Microglia have been implicated in the early stages of corticostriatal synaptic loss and cognitive dysfunction. To shed light on the role of microglia in mediating executive dysfunction induced by exogenous LPC in the medial prefrontal cortex (mPFC), we investigated the phenotypic changes in microglial activation. Immunofluorescence staining of the inflammatory marker MHC‐II (Figure 2G,I, *n* = 8, *t* = 3.859, *p* = 0.0023) and the lysosomal marker CD68 (Figure 2H,I, *n* = 12, *t* = 6.620, *p* < 0.0001) revealed substantial microglial activation, indicating involvement in both phagocytosis and inflammation following LPC injection. This state is associated with neuroinflammatory responses and may exacerbate neuronal damage. This observation was further validated in vitro using the BV2 microglial cell line, where LPC triggered a significant upregulation in the expression of inflammatory molecules ((Figure 2J COX‐2, *n* = 3, *t* = 2.297, *p* = 0.0031), TNF‐α (*n* = 3, *t* = 7.502, *p* = 0.0065), iNOS (*n* = 4, *t* = 4.913, *p* = 0.2648)), along with transcriptional activation of microglia at 48 h, as confirmed by qPCR experiments (Figure 2K). Subsequently, we conducted further validation using primary microglial cells. This involved verifying the expression of inflammatory proteins, such as TNF‐α, COX‐2, and iNOS, through Western blotting (WB) and confirming alterations in gene expression following LPC treatment using quantitative polymerase chain reaction (qPCR) (Figure S4A–C).

**VIDEO 1:** Rotrod test. Video content can be viewed at https://onlinelibrary.wiley.com/doi/10.1111/acel.70003

**VIDEO 2:** Open field test (MPTP). Video content can be viewed at https://onlinelibrary.wiley.com/doi/10.1111/acel.70003

**VIDEO 3:** Open field test (Saline). Video content can be viewed at https://onlinelibrary.wiley.com/doi/10.1111/acel.70003

**VIDEO 4:** Gait test (MPTP). Video content can be viewed at https://onlinelibrary.wiley.com/doi/10.1111/acel.70003

**VIDEO 5:** Gait test (Saline). Video content can be viewed at https://onlinelibrary.wiley.com/doi/10.1111/acel.70003

In summary, the introduction of LPC induces activation of microglia in the mPFC, engaging both inflammatory responses and phagocytic processes. This underscores the need for further exploration into how these microglial activities contribute to the development of executive dysfunction.

### 3.3 Microglial Activation Mediated by LPC Instigates the Engulfment of Dopaminergic Presynaptic Components

**FIGURE 3:**
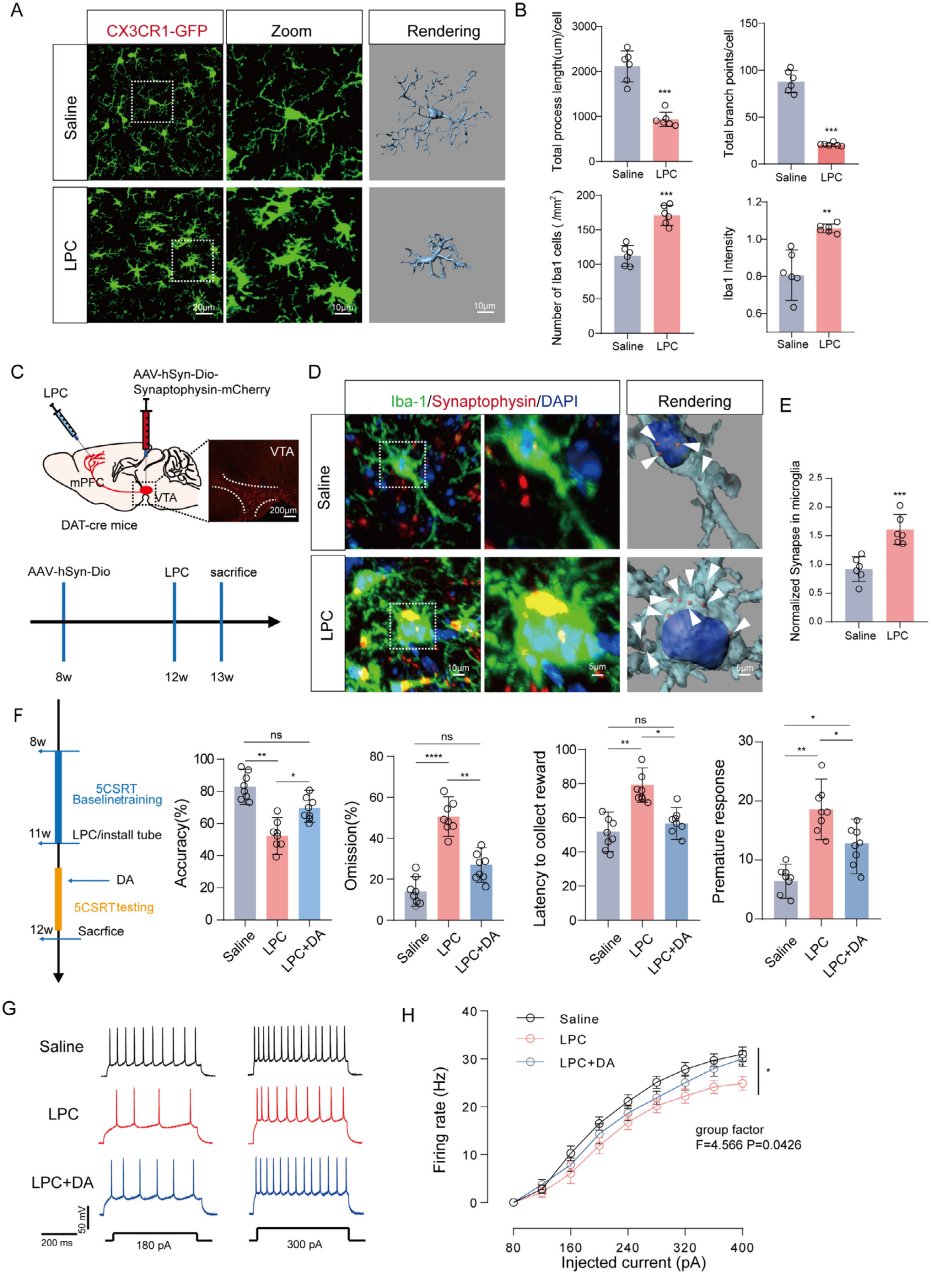
LPC‐mediated microglia activation drives engulfment of dopaminergic presynaptic component and results in a reduction in neuronal activity within mPFC. (A) Fluorescent staining images of CXCR1‐GFP mouse mPFC microglia Iba1 after injection with LPC and Saline, enlarged image of single cell and 3D rendering image. (B) The average branch length of a single cell, the average sub‐count, the number of Iba1 positive cells, and the immunofluorescence intensity of Iba1 positive cells. (C) Schematic diagram of DAT‐Cre mouse VTA injection with anterograde tracer virus. (D) Immunofluorescence and three‐dimensional rendering of contact between IBa1‐positive microglia (green) and mPFC dopaminergic neuron endings. (E) Statistics of the contact area between microglia and synaptic terminals of neurons. (F) The behavioral flow diagram of 5CSRT was performed after LPC treated mice were supplemented with exogenous DA agonists. (G) Statistical accuracy rate (*n* = 8), error rate, delayed reward and immature response of mice in Saline group, LPC group and LPC + DA group of 5CSRT touch screen behavioral results. (H) Statistical plots of representative Traces and firing frequency of action potentials of mPFC pyramidal neurons (*n* = 6). In all statistical results, each point represented one mouse, and all data were expressed as mean ± SEM, *p < 0.05, **p < 0.01, ***p < 0.001, ****p < 0.0001, ns represented no significant difference.

The manifestation of executive dysfunction may arise from abnormal dopaminergic signal loss of mPFC (Zhou et al. 2024). However, the dopaminergic synapse loss in the mPFC induced by microglia in LPC‐mediated executive dysfunction remains insufficiently explored. To investigate the potential abnormal activation of microglia and their phagocytosis of dopaminergic synapses after LPC injection, we employed *CX3CR1‐GFP* reporter mice, specifically labeling microglia in the mPFC. Microglial activation can be broadly categorized into pro‐inflammatory (often referred to as M1‐like) and anti‐inflammatory or reparative (M2‐like) phenotypes. Upon activation, microglia adopt an amoeboid shape, with retracted processes and an enlarged soma, indicative of increased phagocytic activity. Our observations revealed significant microglial activation (Figure 3A) characterized by increased numbers (*n* = 6, *t* = 6.876, *p* < 0.0001), enlarged cell bodies (*n* = 6, *t* = 4.474, *p* = 0.0012), reduced branching (*n* = 6, *t* = 13.92, *p* < 0.0001), and shorter branch lengths (*n* = 6, *t* = 7.570, *p* < 0.0001) (Figure 3B). Using antegrade tracer viruses recognizing cre in the ventral tegmental area (VTA) of *DAT‐cre* mice, targeting dopamine neuron synaptic terminals projecting to the VTA‐mPFC (Figure 3C), we assessed microglial phagocytosis post‐LPC injection. Immunofluorescence staining of IBA‐1 positive cells confirmed that microglia in the mPFC of LPC‐injected mice contained a higher density of dopamine projection terminals (*n* = 6, *t* = 4.993, *p* = 0.0005) (Figure 3D,E). The suggestion is that the pivotal factor in LPC‐induced executive dysfunction in mice involves abnormal microglial activation and heightened phagocytosis of dopamine neuron terminals.

### 3.4 Subsequent Decreased Activity in the mPFC Following LPC Intervation, Accompanied by Dopamine‐Rescued Behavior and Firing Activity

The dopamine content in the prefrontal brain region is vital for maintaining normal basic field neuronal activity. We confirmed the key role of VTA‐mPFC in executive function in mice through photogenetic experiments and dopamine probes, and the injection of LPC can lead to a decrease in the release of dopamine transmitter in the mPFC brain region (Figure S1). Our findings confirm that LPC induces excessive phagocytosis of dopamine terminals in the mPFC by microglia, potentially diminishing dopamine levels and disrupting normal physiological function. We intraperitoneally injected mice with dopamine agonists to elevate dopamine content. Subsequently, executive function, assessed through 5‐CSRT touchscreen behavior, notably improved (Figure 3F). Evaluation indicators: accuracy 70% (LPC + DA) 50% (LPC) (LPC vs. LPC + DA, F = 9.468, *p* = 0.0258, Saline vs. LPC, *p* = 0.0026), omission 30% (LPC + DA) 50% (LPC) (LPC vs. LPC + DA, F = 23.65, *p* = 0.0024, Saline vs. LPC, *p* < 0.0001), latency to collect the reward 55 (Saline) 75 (LPC) (LPC vs. LPC + DA, F = 9.983, *p* = 0.0123, Saline vs. LPC, *p* = 0.0034), and premature response 10 (Saline) 20 (LPC) (LPC vs. LPC + DA, F = 9.188, *p* = 0.0225, Saline vs. LPC, *p* = 0.0028) (Figure 3F), demonstrated improvement. Pyramidal neuronal activity in the mPFC was examined using brain tablet patch clamp, supplemented with exogenous dopamine. Our results indicated that the frequency distribution of action potentials in mPFC pyramidal neurons decreased post‐LPC injection and significantly recovered after restoring dopamine (F = 4.566, *p* = 0.0428) (Figure 3G,H). This underscores the crucial role of dopamine in maintaining neuronal activity and triggering executive function. We posit that excessive phagocytosis of dopamine endings, disrupting normal dopamine release, is a key factor in the onset of executive dysfunction.

### 3.5 Stimulation by LPC Triggers the Activation of Complement Signals Responsible for Synaptic Clearance in Dopaminergic Terminals

**FIGURE 4:**
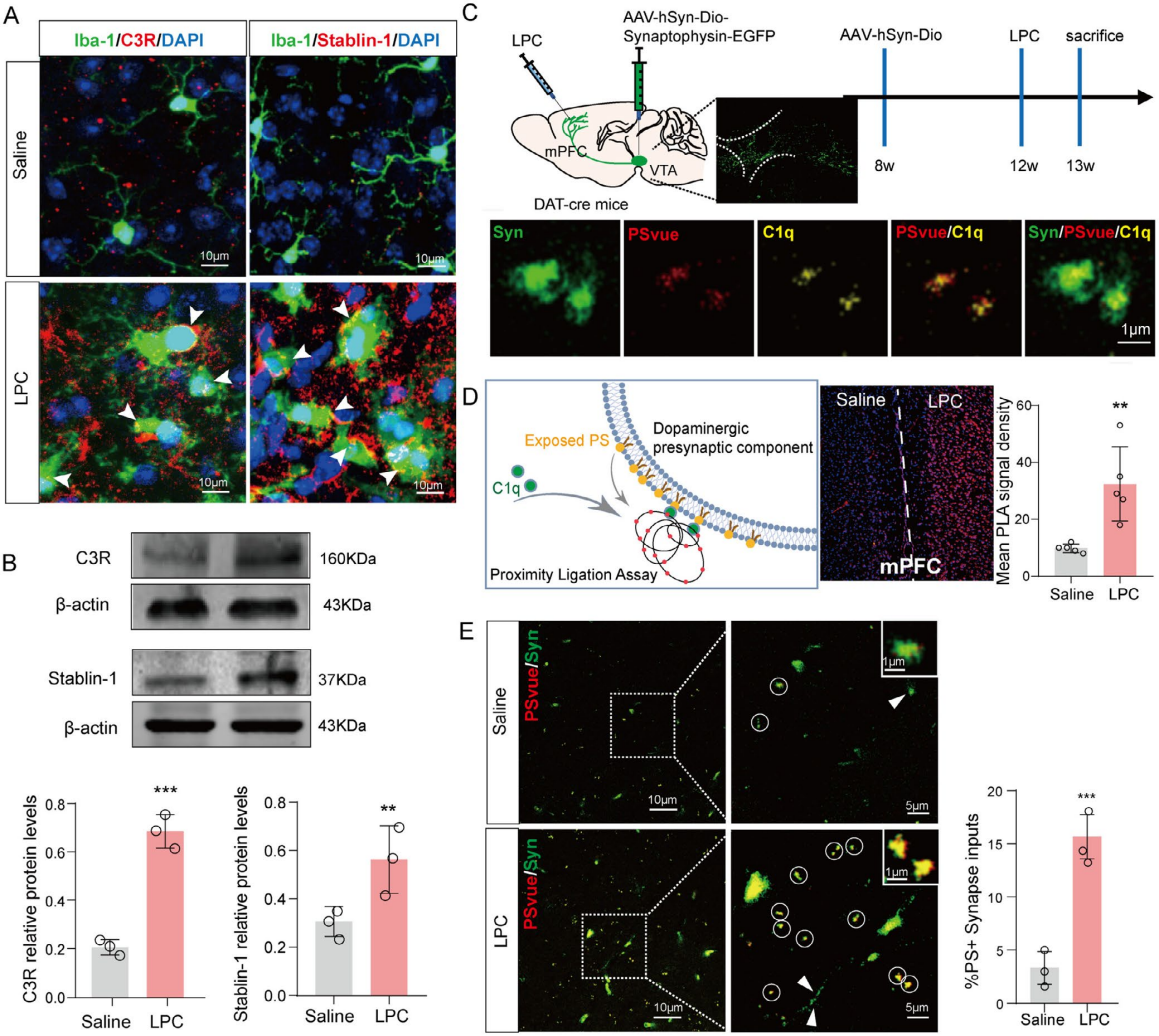
Activation of synaptic clearance complement signals in dopaminergic terminals under LPC stimulation. (A) Immunofluorescence co‐labeling of phagocytic receptor markers C3R and STAB1 with Iba1 and DAPI. (B) Protein Western blot bands and expression level statistics of C3R and STAB1. (C) Schematic diagram and flow diagram of retrograde tracer virus injection in *DAT‐cre* mice. Synapse terminal marker Syn, PS probe and C1q immunofluorescence co‐mapping. (D) PLA fluorescence map and statistics of PS/C1q signal after LPC injection (E) Co‐immunofluorescence map and statistics of PS labeled by PS probe and DA energy axon endings displayed by tracer virus. In all statistical results, each point represented a mouse, and all data were expressed as mean ± SEM, *p < 0.05, **p < 0.01, ***p < 0.001, ****p < 0.0001.

It has been reported that complement proteins play a crucial role in selectively eliminating synapses during the early stages of disease pathogenesis (Wilton et al. 2023). In the mPFC brain region following LPC intervention, the fluorescence results revealed a notable co‐localization between the membrane of Iba‐1‐labeled microglia (green) and the positive regions expressing C3R (red) and Stablin‐1 (red), with a percentage ranging from 15% to 20% (Figure 4A). The involvement of the C3R protein in synaptic pruning as a receptor for C1q signaling was confirmed, as evidenced by our Western blot (WB) results showing an increase in the expression of phagocytic receptor‐related proteins after LPC treatment compared to the Saline group (*n* = 3, *t* = 10.85, *p* = 0.0004, *n* = 3, *t* = 2.900, *p* = 0.0441) (Figure 4B). Further examination in the prefrontal brain region of LPC‐treated mice revealed a significant presence of valgus PS signals alongside DA axon endings. Recognizing that PS exposure indicates early cell function weakening and is a distinct characteristic of apoptosis, and considering PS as a key ligand of apoptotic cells, which can be identified and cleared by macrophages, we hypothesized the involvement of the C3R‐C1q‐PS signaling pathway in mediating microglia‐induced abnormal pruning of DAergic endings. To validate this hypothesis, we conducted experiments injecting the anterograde tracing virus into the VTA region to label mPFC brain DA axon endings (green). Utilizing PS probe and immunofluorescence technology to visualize exposed PS (red) and marking C1q positive (yellow), we observed co‐localization of the three elements (Figure 4C). To substantiate the interaction, the PLA‐doulink assay indicates a heightened binding of PS and C1q in the mPFC region, leading to an augmentation of the PLA signal (*n* = 5, *t* = 3.853, *p* = 0.0049) (Figure 4D). Finally, we noted a substantial increase in PS exposure following the administration of LPC in the mPFC (*n* = 3, *t* = 8.273, *p* = 0.0012) (Figure 4E). In summary, synaptic clearance complements signaling formation, and the expression of complement signal receptors increases in microglia. Additionally, dopaminergic terminals of mPFC generate phagocytic signals.

### 3.6 Artificial Inhibition of mPFC Microglia Holds the Potential to Ameliorate the Executive Dysfunction Behavior Observed in LPC‐Treated Mice

From the aforementioned findings, it is evident that LPC induces executive dysfunction in mice, attributed to the involvement of microglial cells in aberrant pruning of dopaminergic endings in the VTA‐mPFC. To precisely modulate microglia, we employed a chemical genetic approach to construct DREADDS (Designer Receptors Exclusively Activated by Designer Drugs) carrying hM4Di on microglia, utilizing the toxic receptor and CNO as its ligand to mediate inhibited microglial activation.

**FIGURE 5:**
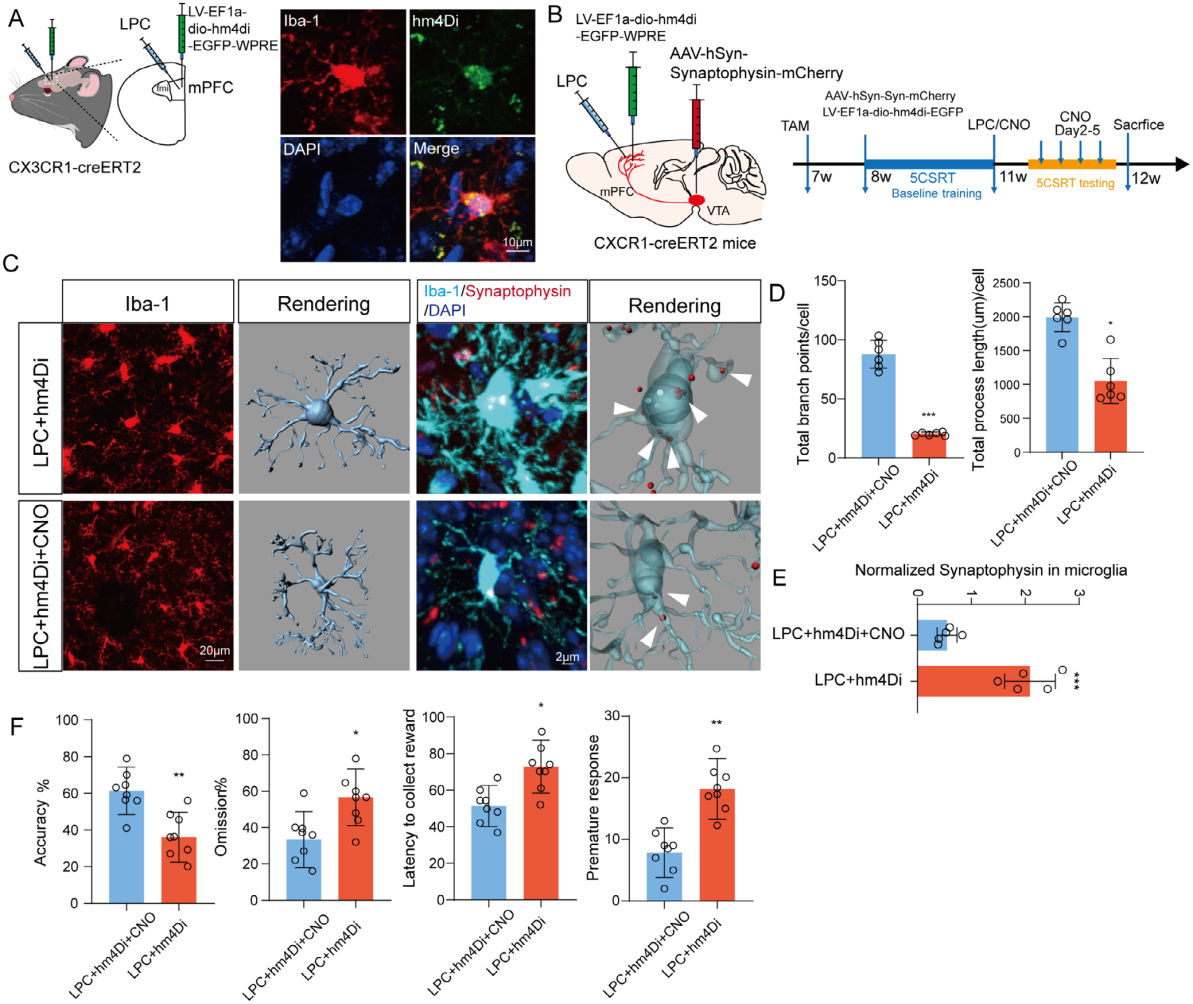
Chemogenetic inhibition of LPC‐induced microglia activation reduces the phagocytosis of dopaminergic endings and behavioral abnormalities. (A) Model map and immunofluorescence map of unilateral injection of chemogenetic inhibitory virus hM4Di in mPFC brain region of *Cx3cr1‐CreERT2* mice. (B) Flow diagram of behavioral detection of 5CSRT after chemogenetic inhibition of LPC‐activated microglia. (C) Immunofluorescence image of mouse mPFC microglia Iba1 after hM4Di virus injection and three‐dimensional rendering of single cell after enlargement. Immunofluorescence and 3D rendering of contact between IBa1‐positive microglia (blue) and mPFC dopaminergic neuron endings. (D) The average branch length of a single cell and the average number of branches. (E) Statistics of the contact area between microglia and synaptic terminals of neurons. (F) Behavioral accuracy, error rate, reward delay and immature response of mice injected with chemogenetic inhibitory virus 5CSRT. In all statistical results, each point represented a mouse, and all data were expressed as mean ± SEM, *p < 0.05, **p < 0.01, ***p < 0.001, ****p < 0.0001.

To achieve controlled microglial manipulation, we utilized *Cx3cr1‐ Cre* ^*ERT2*^ mice with VTA injections of anteroviral constructs after intraperitoneal tamoxifen administration, confirming the expression of DA‐ergic axon terminals. Additionally, chemogenetic inhibitory virus injections were performed in the mPFC brain region using LV‐EF1a‐dio and hM4Di‐EGFP‐WPRE constructs to meet testing criteria (Figure 5A). Mice were then subjected to localized LPC injection in the mPFC brain region, concurrent intraperitoneal CNO administration, and continuous CNO administration throughout behavioral testing (Figure 5B).

Immunofluorescence analysis demonstrated successful chemogenetic viral construction on microglia, as evidenced by the colocalization of Iba1‐labeled microglia (green) and red expression from the chemogenetic virus (Figure 5A). The inhibitory effect of the chemogenetic virus on microglial activation by LPC was confirmed, with three‐dimensional rendering further supporting this result (Figure 5C,D) (*n* = 6, *t* = 5.851, *p* = 0.0002) (*n* = 6, *t* = 13.92, *p* < 0.0001). Subsequent examination of DA‐ergic endings revealed a sustained effect after microglial inhibition, confirming microglial involvement in aberrant pruning (Figure 5C,E) (*n* = 5, *t* = 6.818, *p* = 0.0001).

Post‐CNO administration, LPC‐induced executive dysfunction in mice undergoing touchscreen learning significantly improved. The accuracy gradually recovered from 40% to a normal level of approximately 80% (Figure 5F). Key behavioral parameters, including accuracy (60% (LPC + hm4Di + CNO), 40% (LPC + hm4Di) (*n* = 8, *t* = 3.301, *p* = 0.0080)), omission (30% (LPC + hm4Di + CNO), 55% (LPC + hm4Di)) (*t* = 2.609, *p* = 0.0261), latency to collect reward (50 (LPC + hm4Di + CNO), 70 (LPC + hm4Di)) (*t* = 2.871, *p* = 0.0166), and premature response (10 (LPC + hm4Di + CNO), 20 (LPC + hm4Di)) (*t* = 3.986, *p* = 0.0026), all indicated significant improvement after CNO administration compared to the LPC group. In summary, our findings demonstrate a substantial enhancement in executive dysfunction in mice following the inhibition of LPC‐induced microglial activation using chemical genetic methods.

### 3.7 The Initiation of Microglial Activation and the Upregulation of TDP‐43 Are Dependent on miR‐2885 Signaling in the Context of LPC Stimulation

**FIGURE 6:**
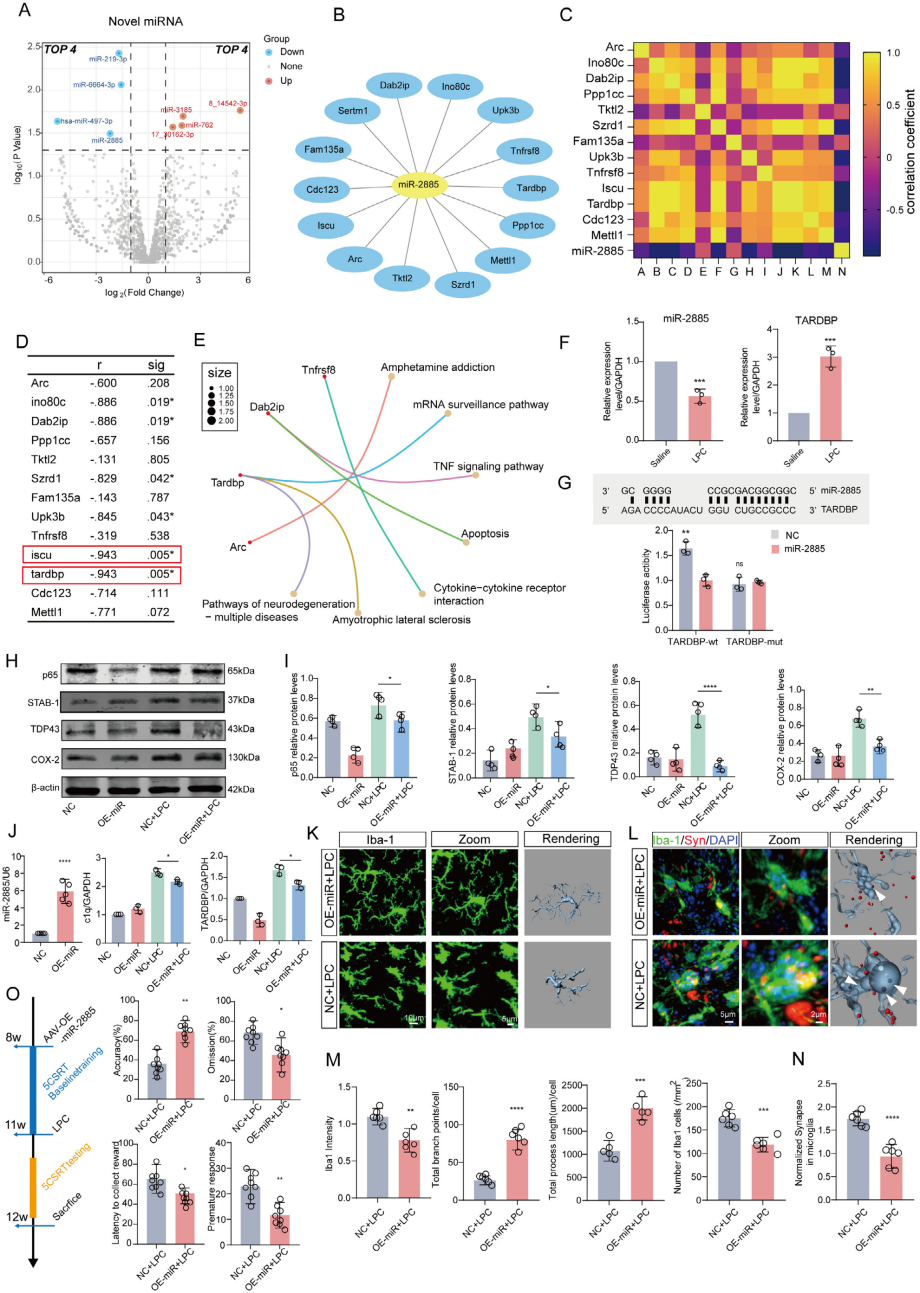
Microglial activation and phagocytosis on dopaminergic terminal via miR‐2885/TDP‐43 signal by LPC stimulation. (A) The heatmap illustrates the differential expression of miRNAs identified through transcriptome sequencing of BV2 microglia treated with LPC, with up‐regulated genes depicted in red and downregulated ones in blue. (B–D) Correlation coefficient statistics of miRNA‐2885 and its associated target genes. (E) Gene Ontology (GO) and Kyoto Encyclopedia of Genes and Genomes (KEGG) analyses elucidate the functional pathways of miRNA‐2885 target genes. (F) Validation of miR‐2885 and its target gene TDP43 through qPCR after BV2 treatment with LPC (*n* = 3, *t* = 8.394, *p* = 0.0011, *t* = 9.258, *p* = 0.0008). (G) Prediction of the binding sequence between miR‐2885 and its target gene TDP43 using the miRbase website. Statistical validation of miR‐2885 and TDP43 binding via dual luciferase reporter experiments (*n* = 3). (H, I) WB assay analysis of protein expression levels (p65, STAB1, TDP43, iNOS, and COX‐2) in BV2 microglia transfected with miR‐2885 virus overexpression after LPC intervention (*n* = 4). (J) qPCR experiment assessing the expression levels of miR‐2885, C1q, and TARDBP genes, with normalization to GAPDH and U6 gene expression. (K) Immunofluorescence, zoom, and 3D rendering depicting Iba1‐expressing microglia in the medial prefrontal cortex (mPFC) of mice injected with miR‐2885 virus overexpression. (M) Statistical analysis of Iba1 fluorescence intensity, microglial branch count and length after rendering, and the number of positive cells. (L, N) Immunofluorescence and statistical analysis of Syn (red) phagocytosis by Iba1‐positive microglia (green) in the mPFC brain region. (O) The 5CSRT touch screen behavior test administered to mice injected with miR‐2885 virus overexpression in the mPFC after LPC intervention. Statistical analysis includes touch screen behavior accuracy, loss rate, delayed reward, and immature response. In all statistical results, each point corresponds to a cell sample or a mouse, and data are presented as average ± SEM. Significance levels are denoted as follows: *p < 0.05, **p < 0.01, ***p < 0.001, ****p < 0.0001, ns represents no significant difference.

We conducted a comprehensive examination of transcriptomic changes in microglial activation associated with LPC‐induced executive dysfunction using RNA‐seq. Microglia isolated post‐LPC and saline injections in the mPFC were subjected to transcriptional analysis (Figure S6). Sequencing showed that there were 1264 up‐regulated differential genes and 1618 down‐regulated ones. Further comparative analysis revealed significant differential expression of miRNAs in LPC‐treated microglia compared to saline‐treated mice. Specifically, miR‐3185, miR‐762, miR‐14,542‐3P, and miR‐30,162‐3P were up‐regulated, while miR‐219‐3P, miR‐6664‐3P, miR‐2885, and miR‐497‐3P were down‐regulated (Figure 6A). Of note, miR‐2885 emerged as a key player, with correlation analysis indicating a prominent association with TDP‐43, encoded by TARDBP (Figure 6B–D). Meanwhile, differential mRNA level analysis also confirmed that TDP‐43 was up‐regulated after LPC (Figure S6D). Abnormal accumulation of TDP‐43, a nuclear DNA/RNA‐binding protein implicated in various neurodegenerative diseases, was observed (Figure 6E). Pathway analysis via Gene Ontology (GO) and Kyoto Encyclopedia of Genes and Genomes (KEGG) implicated TDP‐43 in cellular inflammation and phagocytosis pathways (Figure 6E). This underscores the critical role of TDP‐43 from microglia in maintaining synaptic morphology and function of excitatory neurons in advanced brain regions.

This prompted speculation regarding LPC‐induced downregulation of miR‐2885 in microglia, leading to TDP‐43 overexpression and aberrant synapse pruning. The qPCR results confirmed a nearly 50% reduction in miR‐2885 and a fivefold increase in TDP‐43 expression compared to the control group (Figure 6F) (*n* = 3, *t* = 8.394, *p* = 0.0011, *t* = 9.258, *p* = 0.0008). Further investigations included predicting the miR‐2885 binding site on TDP‐43 through a database website (www.mirbase.org) and conducting dual‐luciferase reporter experiments using miR‐2885, TDP‐43, and TDP‐43 mutant plasmids (TDP‐43 mut). Transfection into BV2 cells demonstrated a tight binding between miR‐2885 and the 3′ UTR of TDP‐43, as evidenced by significantly higher fluorescein intensity compared to the TDP‐43 mut group (Figure 6G) (*n* = 3, *t* = 4.395, *p* = 0.0032, *t* = 1.039, *p* = 0.7513). In summary, the dysregulated microglial activation observed in LPC‐induced executive dysfunction may result from the abnormal downregulation of miR‐2885, leading to heightened TDP‐43 expression and activation of phagocytosis and inflammatory signaling pathways.

### 3.8 The Heightened Expression of miR‐2885 Effectively Mitigates Executive Dysfunction and Impedes the Clearance of Dopaminergic Synapses

We hypothesized that miR‐2885 functions as a pivotal microRNA in the activation of microglia. To investigate this theory, we initially utilized the BV2 microglial cell line and transfected cells with an overexpressed miR‐2885 viral vector (LV‐MIR2885‐up). Western blot results revealed a significant reduction in inflammation‐related proteins, including iNOS (NC + LPC vs. OE‐miR + LPC, f = 7.204, *p* = 0.9907) and COX‐2 (NC + LPC vs. OE‐miR + LPC, f = 19.73, *p* = 0.0023), in the microglia of the miR‐2885 overexpression + LPC group (Figure 6H). This indicates that elevated miR‐2885 levels could mitigate the inflammatory response of microglia. However, a noteworthy decrease in the content of TDP‐43 (NC + LPC vs. OE‐miR + LPC, f = 22.81, *p* < 0.0001), downstream NF‐κB pathway‐related protein p65 (NC + LPC vs. OE‐miR + LPC, f = 20.52, *p* = 0.0220), and phagocytosis‐related protein STAB1 (NC + LPC vs. OE‐miR + LPC, f = 23.62, *p* = 0.0189) was also observed (Figure 6H,I). The qPCR experiments confirmed the consistency of miR‐2885, C1q, and TARDBP gene expression levels with the protein results (Figure 6J) (miR‐2885 (*n* = 3, *t* = 7.803, *p* < 0.0001), C1q (NC + LPC vs. OE‐miR + LPC, f = 119.7, *p* = 0.0303), and TARDBP (NC + LPC vs. OE‐miR + LPC, f = 57.24, *p* = 0.0252)). Collectively, these findings demonstrated that miR‐2885 overexpression significantly improved microglial inflammatory and phagocytosis‐related capabilities, underscoring its critical role in LPC‐mediated microglial activation. Subsequently, in vivo observations of morphological changes in microglia in *Cx3cr1‐CreERT2* mice injected in the mPFC brain region with the miR‐2885 overexpression virus (AAV‐CMV‐DIO‐mCherry‐miR2885) revealed a marked enhancement in microglial activation. This was evident through a significant reduction in the number of microglia (*n* = 6, *t* = 5.729, *p* = 0.0002) compared to the LPC group, accompanied by smaller cell bodies (*n* = 6, *t* = 3.998, *p* = 0.0025) and increased branching (*n* = 6, *t* = 8.985, *p* < 0.0001) (*n* = 6, *t* = 6.083, *p* = 0.0003) (Figure 6M,N). Further, in conjunction with anterograde tracer virus (AAV‐hSyn ‐Synaptophysin ‐mCherry) of VTA‐mPFC, a notable improvement in microglial phagocytosis of DA‐ergic axon terminals and a decrease in the number of red axon terminals within the cells were observed (Figure 6L,N) (*n* = 6, *t* = 6.634, *p* < 0.0001). Analysis of touchscreen behavior revealed that miR‐2885 overexpression in microglia within the mPFC ameliorated executive dysfunction in mice (Figure 6O). This improvement was evident in enhanced accuracy (40% (NC + LPC) vs. 70% (OE‐miR + LPC)) (*n* = 8, *t* = 3.925, *p* = 0.0044), reduced omission (70% (NC + LPC) vs. 40% (OE‐miR + LPC)) (*n* = 8, *t* = 2.414, *p* = 0.0422), delayed latency to collect reward (70 (NC + LPC) vs. 50 (OE‐miR + LPC)) (*n* = 8, *t* = 2.672, *p* = 0.0283), and decreased premature response (25 (NC + LPC) vs. 10 (OE‐miR + LPC)) (*n* = 8, *t* = 2.983, *p* = 0.0175).

### 3.9 Microglial Engulfment of Dopaminergic Terminals is TDP‐43‐Dependent

**FIGURE 7:**
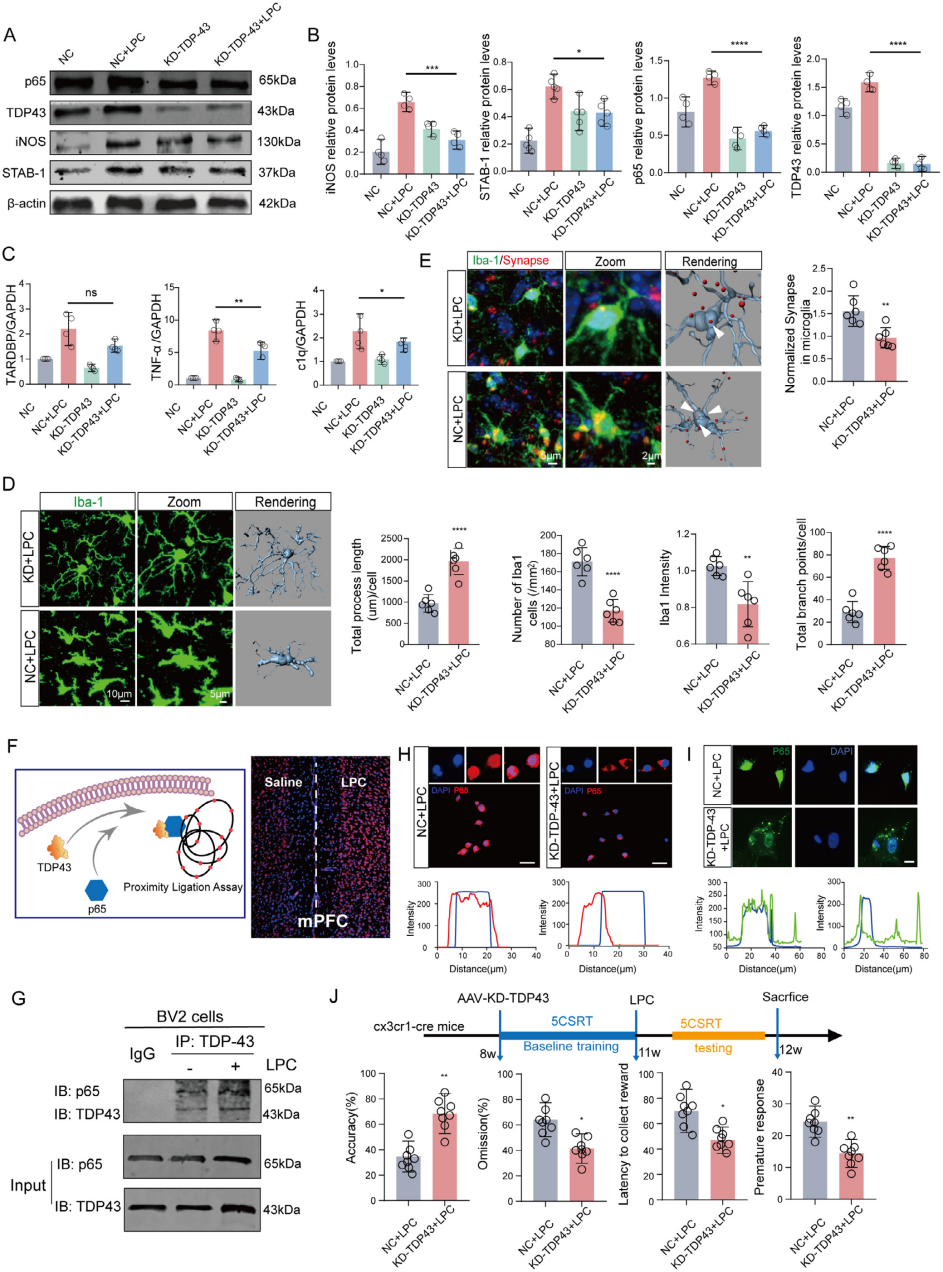
Microglial TDP‐43‐signaling is critical for dopaminergic terminal phagocytosis‐induced executive dysfunction. (A, B) The Western blot (WB) assay was employed to assess the protein expression levels of p65, STAB1, TDP‐43, and iNOS in BV2 microglia following transfection with the TDP‐43 knockout virus in the context of LPC treatment (*n* = 4). (C) Quantitative polymerase chain reaction (qPCR) analysis was conducted to evaluate the expression levels of TNF‐α (*n* = 3), C1q, and TARDBP genes. Normalization was performed relative to the expression levels of GAPDH and U6 genes. (D, E) Immunofluorescence, zoom, and 3D rendering were employed to depict the Iba1‐expressing microglia in the medial prefrontal cortex (mPFC) of mice injected with the TDP43 knockout virus. Statistical analyses encompassed fluorescence intensity of Iba1, microglial branch count and length, as well as the number of positive cells. Additionally, immunofluorescence and statistical analysis were conducted on Syn (red) phagocytosis by Iba1‐positive microglia (green) in the mPFC brain region. (F) A schematic diagram illustrating the co‐activation of TDP‐43 with P65. Fluorescence maps from proximity ligation assay (PLA) experiments, with the left side serving as the control and the right side injected with LPC. (G) Co‐immunoprecipitation (Co‐IP) bands depicting the interaction between TDP‐43 and P65 in BV2 cells post‐LPC intervention. (H, I) Immunofluorescence map of p65 colocalization with nucleus in BV2 cells and primary microglia. (J) The 5CSRT touch screen behavior test was administered to mice injected with the TDP‐43 virus in the mPFC following LPC intervention. The flow chart depicts the experimental sequence from left to right, and statistical analyses included the correct rate of touch screen behavior, loss rate, delayed reward, and immature response. In all statistical results, each point corresponds to a cell sample or a mouse, with data expressed as the average ± SEM. Significance levels are denoted as follows: *p < 0.05, **p < 0.01, ***p < 0.001, ****p < 0.0001, ns represents no significant difference.

TDP‐43 emerges as a crucial target gene regulated by miR‐2885, playing a pivotal role in the pathogenesis of neurological diseases. This regulatory relationship extends beyond neurons to encompass microglial glia in intercellular communication in our study. In our exploration of the influence of miR‐2885‐regulated TDP‐43 on LPC intervention in mice, we introduced a TDP‐43 knockdown virus into BV2 microglia. Western blot results revealed a significant reduction in TDP‐43 expression upon knockdown, leading to a marked decrease in the expression of inflammatory proteins, including iNOS, in microglia compared to the LPC group. This reduction in inflammatory markers indicated an amelioration of microglial inflammation. Additionally, the expression of the phagocytic‐related protein STAB‐1 was reduced, suggesting an enhancement in microglial phagocytic capacity (Figure 7A,B) p65 (NC + LPC vs. KD‐TDP43 + LPC, f = 27.08, *p* < 0.0001), STAB1 (NC + LPC vs. KD‐TDP43 + LPC, f = 11.51, *p* = 0.0422), TDP43 (NC + LPC vs. KD‐TDP43 + LPC, f = 105.9, *p* < 0.0001), and iNOS (NC + LPC vs. KD‐TDP43 + LPC, f = 18.53, *p* = 0.0007).

Concurrently, qPCR results confirmed the consistency of gene expression levels of TNF‐α, C1q, and TARDBP with the protein results (Figure 7C) (TNF‐α (*n* = 3) (NC + LPC vs. KD‐TDP43 + LPC, f = 45.26, *p* = 0.0070), C1q (NC + LPC vs. KD‐TDP43 + LPC, f = 8.330, *p* = 0.0378), TARDBP (NC + LPC vs. KD‐TDP43 + LPC, f = 13.97, *p* = 0.0958)). Subsequent experiments in mice aimed to validate alterations in touchscreen behavior following TDP‐43 knockdown. In the KD‐TDP 43 + LPC group, microglia numbers and morphology approached levels observed in the normal group (Figure 7D,E) (fluorescence intensity (*n* = 6, *t* = 3.820, *p* = 0.0034), number of microglia branches after rendering (*n* = 6, *t* = 8.529, *p* < 0.0001) and length (*n* = 6, *t* = 6.534, *p* < 0.0001), statistical analysis of the number of positive cells (*n* = 6, *t* = 6.649, *p* < 0.0001)). Notably, a significant reduction in the engulfment of DAergic endings (Figure 7D,E) (*n* = 6, *t* = 3.542, *p* = 0.0053) was observed. TDP‐43 serves as a co‐activator for the p65 protein, facilitating downstream transcription. The outcomes of the proximity ligation assay (PLA) experiment revealed a notable augmentation in fluorescence within the right medial prefrontal cortex (mPFC) brain region following LPC injection, indicative of an increased binding between TDP‐43 and P65 (Figure 7F). Furthermore, cellular‐level co‐immunoprecipitation (Co‐IP) results obtained from BV2 cells and rat microglia also affirmed a heightened co‐activation of TDP‐43 and P65 subsequent to LPC intervention (Figure 7G). We conducted Western blot (WB) experiments to compare the levels of P65 protein in both the cytoplasm and nucleus following LPC treatment. Additionally, we performed immunofluorescence staining to observe the nuclear translocation of P65 after LPC exposure (Figure S5A,B). We found that p65 enucleation was significantly reduced after selective removal of TDP‐43 from microglia following LPC treatment, which implied that TDP‐43 acts as a co‐activator of p65 to help p65 enter the nucleus and regulate transcription (Figure 7H). The 5‐CSRT touchscreen behavior demonstrated marked improvement, with mice displaying significantly enhanced accuracy (30% NC + LPC vs. 70% KO‐TDP‐43 + LPC) (*n* = 8, *t* = 3.802, *p* = 0.0052), reduced omission (60% NC + LPC vs. 40% KO‐TDP‐43 + LPC) (*n* = 8, *t* = 2.879, *p* = 0.0205), decreased latency to collect reward (70 NC + LPC vs. 40 KO‐TDP‐43 + LPC) (*n* = 8, *t* = 2.586, *p* = 0.0323), and diminished premature responses (25 NC + LPC vs. 15 KO‐TDP‐43 + LPC) (*n* = 8, *t* = 3.386, *p* = 0.0096) (Figure 7I). The cumulative results imply that suppressing TDP‐43 has a beneficial impact on alleviating the adverse effects of LPC. This occurs through the correction of abnormal microglial pruning of dopaminergic (DAergic) endings, ultimately leading to an improvement in executive function in mice.

## 4 Discussion

In this study, we combined clinical serum LPC detection, cognitive assessment, and experimental techniques such as three‐dimensional (3D) reconstruction, virus tracing, PLA‐doulink, electrophysiology, and DREADD‐based approaches for manipulating microglial activity. Initially, we verified the abnormal elevation in serum LPC levels observed clinically in PD patients, establishing a significant negative correlation with cognitive function. Subsequently, in our PD mouse model, we observed a notable exacerbation of executive dysfunction when microglia in the mPFC were exposed to LPC. Moreover, we found that LPC injection into the mPFC induced a dose‐dependent and rapid onset of microglial activation, which spatially correlated with dopaminergic terminal degeneration in normal wild‐type mice. Importantly, LPC intervention alone in the mPFC was adequate to induce executive dysfunction. Furthermore, we noted that the phagocytosis of dopaminergic terminals was reversible upon timely inhibition of microglial activity.

While PD ranks as the second most prevalent neurodegenerative disorder, a significant majority, approximately 90%–95%, of PD cases are classified as idiopathic, characterized by an unknown etiology (Armstrong and Okun 2020). Lipids play a crucial role in various aspects of PD pathology, including oxidative stress and inflammation. Dysregulation of lipids or disturbances in lipid homeostasis may potentially contribute to the pathogenesis of this disease (Dahabiyeh et al. 2023). Our prior research supported significant differences in the proteomic and metabolic profiles of PD, revealing inflammatory and lipid metabolism profile alterations. Specifically, LPC (18:1) levels were markedly elevated, showing a strong negative correlation with cognition scores. Additionally, LPC (18,1) demonstrated potential clinical value in distinguishing the presence or absence of cognitive impairment in PD (Zhang et al. 2021).

Furthermore, a study unveiled abnormal alterations in lipid substances, particularly the abnormal elevation of LPC (16,0) and (18,1), by utilizing a low dose of the specific neurotoxin 6‐hydroxydopamine (6‐OHDA) to induce a prodromal‐like PD state (Farmer et al. 2015). These findings represent a crucial advancement in understanding lipid changes in the early stages of PD‐like pathology. Another study revealed a significant increase in LPC content in the cerebrospinal fluid during the pathological stage of PD (Li et al. 2023). Certainly, environmental and systemic factors, including gut‐brain axis involvement, likely play a significant role in influencing LPC levels. The gut dysbiosis can lead to increased intestinal permeability and potentially elevate circulating LPC levels (Zha et al. 2025). Additionally, disruptions in lysophosphatidylcholine acyltransferase 1 (LPCAT1), the enzyme that regulates LPC content, could impact the aggregation of α‐syn pre‐formed fibrils. Importantly, LPC has the potential to interact with α‐syn, promoting synaptic vesicle clustering and influencing the conformational dynamics of α‐syn in neurons (Lai et al. 2023). Hence, in this study, we have substantial grounds to suggest that the impact of LPC on the mPFC may mimic the progression of neurodegeneration and the decline in executive function during the early stages of PD.

PD‐EDF emerges as a pivotal non‐motor symptom, with the mPFC serving as the central hub for governing executive function in the brain. Despite constituting only approximately 0.01% of neurons in the brain, those responsible for DA production wield significant influence over a diverse array of brain processes. Specifically, dopamine in the mPFC plays a crucial role in sustaining executive function, regulating reward systems, and influencing learning and memory (Liu et al. 2018). Previous studies have proposed that the diminishment of dopaminergic axon endings within mPFC regions could serve as a crucial instigator for the onset of executive dysfunction (Tang et al. 2023; Zhou et al. 2024). These findings strongly suggest that the depletion of DA axon endings is a causal factor in the onset of PD‐EDF. In our additional analysis process, we also took into account the impact of gender on executive function. The results indicate that, under the current sample size, there are no significant cognitive differences between males and females in the subgroup (Figure S7). We recognize that larger population‐based studies are probably required to further clarify the relationship between sex and executive dysfunction in PD. In rodent models, the 5‐CSRT, which is widely used to assess executive function in mice, demonstrates no significant sex differences in crucial metrics such as task acquisition, accuracy, omissions, or premature responses (these are the parameters analyzed in our study). Nevertheless, sex differences have been noticed in impulsivity and attention‐related tasks among rodents. Moreover, this comprehensive study concludes that although individual factors may exhibit sex‐biased tendencies (e.g., males tend to have increased impulsivity and reduced reaction times, while females often have better working memory), the overall differences in executive function between the two sexes are not particularly prominent, neither in humans nor in animals (Grissom and Reyes 2019). However, this also suggests that in the future, it will be necessary to break down the single components of executive function in mice to take the influence of gender into consideration.

In our study, we have found that LPC significantly influences the activation of microglia, as evidenced by their amoeboid morphology, shortened processes, and enlarged soma. This activation correlates with a heightened loss of dopaminergic terminals in mice, resulting in notable impairments in executive function. Importantly, we have observed that inhibiting microglia can effectively reverse these impairments. Microglia, as the most abundant immune cells within the central nervous system, are believed to play a crucial role in neurodegenerative diseases such as PD (Ho 2019). Activated M1‐like microglia release large amounts of pro‐inflammatory factors that interact with dopaminergic neurons (Wyss‐Coray and Mucke 2002), perpetuating synaptic and neuronal dysfunction. Post‐mortem examination of brain tissue from PD patients has revealed a significant presence of microglia surrounding degenerating dopaminergic neurons (Smajic et al. 2022). The early onset of chronic microglial activation, characterized by the substantial formation of the NLRP3 inflammasome, is pivotal in initiating synaptic pruning and subsequent phagocytosis of dopaminergic synapses (Sierra et al. 2013). This cascade further exacerbates neuronal dysfunction in PD.

Our study revealed that LPC‐induced inflammation led to a reduction in mPFC glutamatergic (mPFC^Glu^) neuronal activity, accompanied by impaired executive‐like behaviors in mice. Interestingly, we observed a notable enhancement in executive function upon dopamine supplementation in these mice. This implies that the functionality of dopaminergic synapses in the mPFC remains intact, crucial for halting the progression of EDF symptoms. These findings suggest that the phagocytosis of dopaminergic synapses by microglia, induced by LPC, may represent an initial pathophysiological event in the neurodegenerative cascade of PD. Moreover, the pathological protein α‐syn, associated with PD, selectively targets microglia, disrupting the autophagolysosomal pathway and amplifying NLRP3 formation (Li, Xia et al. 2021; Qin et al. 2021), thereby sustaining chronic inflammation. Our investigation identifies elevated LPC as a pivotal factor triggering microglial activation, entangling microglia in a detrimental cycle that perpetuates chronic inflammation. Additionally, research suggests that increased serum levels of LPC contribute to an elevated basal inflammatory tone in the human body, indicative of a chronic inflammatory state (Liu et al. 2020; Ismaeel and Qadri 2021).

Our findings demonstrate that LPC‐induced inflammation in 8‐week‐old WT mice significantly activates mPFC microglia, inducing changes in cellular morphology, numbers, and expression of both inflammatory and phagocytic markers. The profound effects of LPC on dopaminergic synapse pruning and executive function behavior in subsequent tests provide a plausible mechanism supporting our previous speculation that LPC acts as a priming factor, driving microglia into a detrimental cycle of “activation‐chronic inflammation.” Furthermore, our study substantiates that employing chemogenetic methods to inhibit LPC‐induced microglial activation effectively attenuates inflammation. This intervention disrupts the vicious cycle entrapping microglia, resulting in a reduction in both dopaminergic phagocytosis and the decline in executive function. These findings lay a foundational groundwork for understanding and potentially intervening in the complex dynamics of microglial activation and its implications in chronic inflammation associated with PD.

To elucidate the mechanism of microglia activation by LPC, we demonstrated that LPC triggers regional microglia activation via the miR‐2885/TDP43/NF‐κB signal pathway, which subsequently facilitates the engulfment of dopaminergic terminals in the mPFC mediated by the “eat me” signal. C1q acts as the initiator in the classical complement cascade, playing a pivotal role in guiding microglia toward the early phagocytosis of synapses (Hong et al. 2016). Dysfunction of axon endings, characterized by neuronal tau aggregation (Brelstaff et al. 2021), mutation of the turnover enzyme CDC50A (Li, Yu et al. 2021), and less active synaptic connections (Li et al. 2012), exposes the “eat me” signal phosphatidylserine (PS), leading to subsequent pruning by phagocytic microglia (Scott‐Hewitt et al. 2020; Soteros and Sia 2022). In our study, the dopaminergic endings were no exception, as we observed a significant increase in PS exposure in the DA energetic endings of the mPFC following LPC stimulation.

Moreover, these endings generated phagocytic signaling ligands interacting with complement C1q. Our investigation unveiled elevated levels of both the classic synapse phagocytic receptor C3R (Stevens et al. 2007) and the recently identified STAB‐1 receptor (Park and Kim 2019), indicating their involvement in the observed phagocytosis. We discovered that miR‐2885 was downregulated, thereby enhancing the expression of TDP‐43. It acts as a facilitator, assisting the transcription factor P65 in nuclear entry. Upon LPC stimulation, the “TDP43/P65” complex exhibits an augmentation. However, the nuclear entry of P65 is hampered, impeding complex formation, attributable to decreased TDP43 expression. The NF‐κB signal serves as a crucial transcription factor essential for microglia, promoting the release of inflammatory factors and the expression of phagocytic receptors (Bras et al. 2020; Wei et al. 2023). As a versatile nucleic acid‐binding protein, TDP43 plays a wide role in regulating gene expression in cells. Consistent evidence suggests that glial cells expressing higher levels of TDP43, acting as a co‐activator of P65, demonstrate increased production of proinflammatory cytokines and neurotoxic mediators when stimulated by lipopolysaccharide or reactive oxygen species (Swarup et al. 2011). Furthermore, in the zebrafish stab injury model, a direct correlation was observed between the degree of microglia activation and the accumulation of TDP43 condensates (Zambusi et al. 2022). These collective findings imply that microglial activation, along with the phagocytosis of dopaminergic endings following LPC stimulation and the upsurge of TDP43 levels in microglia, constitute pivotal mechanisms propelling the activation process.

The molecular mechanisms that govern “Neuron–Glia” signaling in synapse elimination are still not fully understood. First, we need further clarification on the attenuation of mPFC synaptic transmission in dopaminergic synapses following LPC stimulation and the mechanism underlying PS exposure in dopaminergic (DAergic) endings. Second, we need to investigate how LPC activates microglia. Our preliminary sequencing analysis suggests the involvement of GPR120 in LPC signal transduction, which is consistent with recent research identifying LPC as activating ligands for the orphan receptor GPR119 (Xu et al. 2022). This association warrants deeper exploration in future studies. Additionally, our present study suggests that LPC does not have a region‐specific impact on the abnormal activation of microglia. Given the crucial role of the DA system within the mPFC in executive function, we intentionally intervened in the mPFC using LPC. Moving forward, it is essential to examine the correlation between LPC levels in serum and different brain regions over time. It is also important to determine whether LPC elicits distinct activation effects on microglia with different phenotypes. Furthermore, future research efforts will focus on understanding microglia sensitivity to dopamine, particularly whether these microglia exhibit specificity and uniqueness in response to DA system degradation.

**FIGURE 8:**
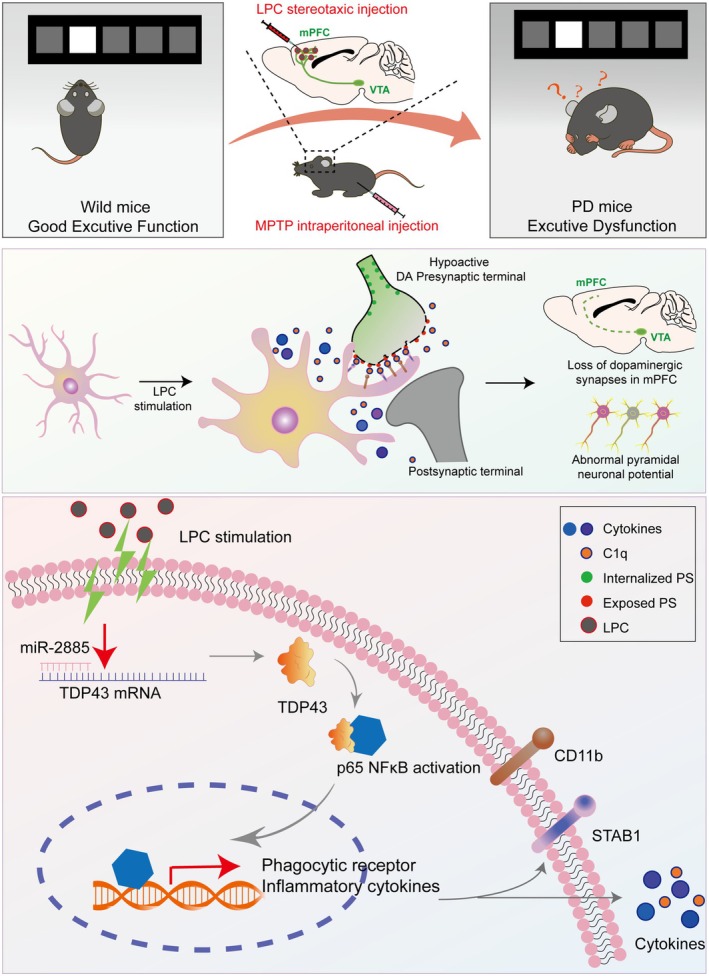
Graphical Abstract: Parkinson's disease with executive dysfunction is a challenge. Our study reveals a novel pathway where abnormally elevated LPC induces miR‐2885/TDP‐43 signal activation in microglia, leading to dopaminergic terminal phagocytosis, and suggests that supplementing dopamine or inhibiting related microglial activation can mitigate the dysfunction, providing insights for potential therapies.

In summary, our study (Figure 8) demonstrates that acute LPC exposure in 8‐week‐old mice induces microglial activation via the miR‐2885/TDP43/NF‐κB signaling pathway in the mPFC. This activation leads to the upregulation of C3R, Stab‐1, and proinflammatory cytokines such as COX‐2 and TNF‐α, as well as a reduction in mPFC^Glu^ neuronal excitability, resulting in executive dysfunction. The LPC‐induced microglial activation promotes excessive engulfment of DAergic endings by triggering “eat me” signals, which further decreases mPFC^Glu^ neuronal activity. Suppression of microglial activity or dopamine supplementation effectively reverses these effects and improves executive dysfunction in mice. Our study suggests that LPC‐induced microglial activation contributes to the degeneration of dopaminergic terminals in the mPFC, leading to executive dysfunction in PD. Additionally, we hypothesize that LPC stimulation may increase the population of disease‐associated microglia (DAM) phenotype, thereby exacerbating the cycle of “activation‐chronic inflammation.” These findings underscore the importance of mPFC microglia in maintaining dopamine homeostasis and provide new insights into the role of LPC as a risk factor in the progression of executive dysfunction in PD.

### 4.1 Study Approval

The clinical study was performed in accordance with Declaration of Helsinki principles. All clinical samples collected were approved by the Ethics Committee of the Affiliated Hospital of Xuzhou Medical University (Approval No. XYFY2017‐KL047‐01). Written informed consent was received by all patients prior to enrollment in the study. All animal study was approved by and performed in accordance with the guidelines of the IACUC of Xuzhou Medical University (Experimental Animal Ethics number: 202209S042).

## Author Contributions

C.X. and X.L. conceived the project and designed the study; C.X. and Y.H. wrote the manuscript; C.R., C.Y., J.J., Y.R., S.J., X.Y., and C.X. performed the experiments and acquired animal research data; J.J., X.Y., S.J., and M.Y. provided scientific input and English‐editing work; C.R. and Y.R. performed clinical peripheral blood sample collection and cognitive evaluation from healthy subjects and PD patients. C.X., Y.H., C.R., C.Y., M.Y., and Z.Z. contributed to analysis.

## Conflicts of Interest

The authors declare no conflicts of interest.

## Supporting information


File S1.

File S2.

File S3.

Data S1.

## Data Availability

All data are available in the main text or the Supporting Information.
