# Supplementary material for: Enhanced Microglial Engulfment of Dopaminergic Synapses Induces Parkinson's Disease‐Related Executive Dysfunction in an Acute LPC Infusion Targeting the mPFC
Source: Aging Cell. 2025 Feb 15;24(5):e70003. doi: 10.1111/acel.70003 (PMC12073916; doi:10.1111/acel.70003)
Supplement: Supplementary file 2 — File S2. [file ACEL-24-e70003-s002.docx]

**EXPERIMENTAL MODEL AND SUBJECT DETAILS**

**Participants**

Participants were recruited from the Affiliated Hospital of Xuzhou Medical University under the approval of the Ethics Committee (Approval No. XYFY2017-KL047-01). Inclusion criteria involved individuals diagnosed with Parkinson's disease by two neurologists, meeting the Parkinson's Disease UK Brain Bank criteria, and having no history of neurosurgical treatment. Exclusion criteria included non-idiopathic Parkinson's disease, Lewy body dementia, severe brain injury, and significant medical conditions (e.g., heart failure, mental illness, malignancy), as well as cognitive impairment hindering informed consent. Age- and sex-matched healthy control individuals were enlisted from the spouses and friends of Parkinson's disease participants. Blood samples were collected from all participants for testing purposes. Cognitive assessments utilized various scales, including the Mini-Mental State Examination (MMSE), the Montreal Cognitive Assessment (MoCA), and the Trail Making Test-B (TMT-B), to evaluate specific cognitive domains.

**Animals**

Considering the gender prevalence of PD incidence, there were more males than females, and the universality of MPTP modeling. So we chose male mice and MPTP modeling method. The 8-week-old male C57BL/6J mice utilized in this study were sourced from the Experimental Animal Center of Xuzhou Medical University. *DAT-Cre* mice, carrying license No. SCXK (Su) 2018-0003, were procured from Saiye Model Biological Research Center Co., LTD. Additionally, *Cx3cr1-GFP* and *Cx3cr1-Cre^ERT2^* mice were generously provided by Professor Wang Haitao's research group. Adherence to ethical guidelines set by the Xuzhou Medical University Animal Care and Use Committee was strictly maintained (Experimental Animal Ethics number: 202209S042). All mice were housed in an environment with a controlled temperature range (23-25°C) and subjected to a regular 12-hour light-dark cycle. The experimental cohort consisted exclusively of male mice aged 8-10 weeks. Throughout the duration of the study, mice enjoyed unrestricted movement and had continuous access to both food and water. In our experimental procedures, meticulous care was taken to ensure the judicious use of all mice. Some individuals were excluded from the study due to inaccuracies in injection positions during surgical interventions, and these instances were consistently reflected in the exclusion of corresponding data from the overall experimental results.

**METHOD DETAILS**

**Drug administration**

For mice demonstrating a DA response, an intraperitoneal injection of the DA agonist SKF 38393 hydrochloride (Cat#SKF-38393A, MedChemExpress,) was administered at a dosage of 10 mg/kg.

Clozapine-N-oxide (CNO), sourced from Sigma-Aldrich (Cat#C0832), was initially dissolved in DMSO at a concentration of 10 mg/ml as a stock solution. For DREADDs experiments, it was further diluted in 0.9% saline and injected at a dose of 5 mg/kg intraperitoneally, 30 minutes prior to behavioral tests. Tamoxifen (TAM), obtained from Sigma-Aldrich (Cat#T5648), was initially dissolved in 100% ethanol at a concentration of 20 mg/ml as a stock solution. To create a final concentration of 2 mg/ml, it was then suspended in corn oil (Cat#C8267, Sigma-Aldrich). In the case of *Cx3cr1-Cre^ERT2^* mice, after virus injection, a total of 50 mL of the 2 mg/ml TAM solution was injected into mice over four consecutive days to activate Cre activity.

**PD models**

The MPTP-induced PD mouse model is one of the most widely used and classic models of PD. It effectively replicates motor symptoms of PD, particularly dopaminergic neuronal damage and motor dysfunction. This model induces pathological changes similar to human PD by selectively damaging midbrain dopaminergic neurons. Compared to other models, the MPTP model offers excellent controllability, allowing researchers to adjust the degree of neuronal damage through different dosages and administration protocols, thereby facilitating more flexible experimental designs. A substantial body of prior research has utilized the MPTP model, accumulating rich experimental data and clinical relevance. On the whole, The MPTP - induced mouse model of PD now stands out among the other toxin models in PD research (Blandini and Armentero 2012, Mustapha and Mat Taib 2021).

In this investigation, male C57BL/6J mice weighing between 23-25 g underwent intraperitoneal injections of MPTP (7 mg/mL) (Cat# M0896, Sigma, St. Louis, MO, USA) at a dose of 30 mg/kg for five consecutive days, employing the MPTP subacute modeling approach. The Saline group received intraperitoneal injections of an equivalent volume of normal saline.

**Rotarod test**

The motor ability of mice was evaluated using the rotating rod test, which comprised a rotating drum with several separated discs (Zheng Hua Tech Co., Ltd., Hefei, China). Initially, all mice underwent a 30-second adaptation period on the rod spinner. Following this, the test commenced with six mice in each group. Each mouse was positioned on the rod, and the rotation speed increased gradually from 4 rpm to 40 rpm over a span of 300 seconds. Throughout this process, the rod spinner automatically recorded the duration each mouse remained on the rotating rod before dropping.

**Open field test**

An open-field installation (50x50x40 cm) consists of a central square (25x25 cm) and peripheral areas surrounded by high walls. Place your mouse on the corner of the device with 10 lux lighting, explore freely for 5 minutes and record your trajectory. Migration behavior was measured by calculating the total travel distance offline using EthoVision XT software (Noldus).

**Gait imaging**

Mouse gait images were acquired and analyzed using a DigiGait™ imaging system (MouseSpecific, Inc., USA). In the training paradigm, mice were trained on a transparent treadmill three times per day (5 min each time). On the day of the test, the mice were placed on a transparent treadmill, and the working status of the mice was recorded using an imaging system. Statistical analysis of isokinetic mice was performed using DigiGait analysis software.

**Y-maze**

The maze, comprising a central area and three enclosed arms, was positioned on level ground within a tranquil and scent-free environment (Zheng Hua Tech Co., Ltd., Hefei, China). Mice were introduced randomly from a closed arm and granted 5 minutes for unrestricted exploration during the adaptation stage, with only two arms accessible. Subsequently, in the testing phase, all three closed arms were made available, and the time and frequency of entries into the newly accessible open arms were meticulously analyzed. Post each mouse trial, the Y maze underwent thorough cleaning with a solution of alcohol and water to eliminate residual odors and prevent any impact on subsequent mouse assessments.

**Five-item Selective Continuous Reaction Time Task Test (5CSRT)**

The Bussey-Saksida chamber is comprised of an individually tested behavioral chamber for mice, seamlessly integrated with various interconnected components. Configured into a modular polygonal box, the chamber includes features such as lights, sugar and water reward dispensers, touch screen modules, and video recording monitoring devices(Campden Instruments Limited，PO Box 8148，Loughborough，LE12 7XT，UK).

The Bussey-Saksida system represents an advanced cognitive assessment tool tailored for rodents, specifically mice. One of its paradigms, the 5CSRT (5-Choice Serial Reaction Time), provides a precise evaluation of executive function in mice through touchscreen technology. Utilizing sugar water rewards to facilitate behavioral learning, mice are tasked with swiftly identifying a fleeting image flicker within one of five distinct squares on the LCD touchscreen.

In the event of an imprecise touch, an incorrect box selection, or mistimed touch, a brief lighting penalty ensues. Conversely, accurate touches are rewarded with sugar water. Typically, a single experiment comprises 100 tests spanning a 30-minute duration. We have identified motivation as a critical factor influencing task performance, and to control this variable effectively, water deprivation is implemented. Throughout the period of thirst, daily weight monitoring is conducted, and if an animal loses more than 10% of its average weight from the previous two days, it is provided with free water until weight is regained. Similarly, if there is a reduction in water consumption at any point, additional water is made available to the mice.

Upon initiating the 5CSRT task program, mice are individually placed in designated boxes, progressing through learning stages from Session 1 to Session 8. These stages involve familiarizing the mice with touchscreen interaction, refining their ability to touch quickly and accurately, and ultimately, receiving sugar water rewards. Once baseline proficiency is achieved, Session 9 begins, featuring randomly lit modules that mice must touch within a specified time frame. The lighting interval gradually decreases until the mice attain an 80% success rate. Subsequently, drug interventions are introduced, marking the commencement of a five-day test phase.

**Immunohistochemistry, imaging and image Analysis**

Mice were deeply anesthetized with isoflurane, followed by a swift infusion of normal saline and 4% paraformaldehyde for 3 minutes. The brains were then isolated in a 4% paraformaldehyde solution at 4°C, fixed overnight, and subsequently immersed in 20% and 30% sucrose solutions at 4°C, undergoing dehydration for 2-3 days until isoosmotic. Frozen brains were precisely sectioned into 40 μm coronal slices at -20°C using a cryosectioning machine microtome system (CM1860, Leica). These brain slices were immersed in antifreeze and stored at -20°C. For staining, the medial prefrontal cortex (mPFC) brain sections underwent a 5-minute rinse with PBS, followed by closure with 10% normal goat serum at room temperature in PBS with 5% Triton X-100 for 1 hour. Subsequently, the slices were incubated overnight at 4°C with primary antibodies. After a 5-minute PBS rinse, the slices were incubated with secondary antibodies at room temperature in the dark for 1.5 hours. Finally, all brain sections were stained with DAPI dilution buffer (1:2000) for 3 minutes and washed twice. For quantitative immunohistochemical analysis, slide images were captured and visualized using a microscope (CLSM, Leica STELLARIS 5, Germany). Fluorescence intensity quantification of Iba1 protein was conducted at 20 times magnification, with two slices randomly selected from six mice in each group for imaging and quantification. Additional analyses, including cell count and colocalization, were carried out using ImageJ software (ImageJ 1.8.0, National Institutes of Health) by an observer unaware of the experimental conditions. Colocalization intensity was adjusted to accurately count moderately to highly positive immunoreactive cells, and the average count per mouse was used for statistical evaluation.

The antibody dilutions employed were as follows: rabbit anti-Iba1 (1:200, HUABIO), mouse anti-MHC (1:200, HUABIO), mouse anti-CD68 (1:200, HUABIO), mouse anti-CD11b (1:200, HUABIO), mouse anti-C1q (1:200, HUABIO).

**Three-dimensional (3D) reconstruction**

The 40-mm coronal slices underwent anti-Iba1 staining for 24 hours, followed by staining with Alexa Fluor 488-conjugated secondary antibodies. Subsequently, nuclei were counterstained with DAPI. Imaging was conducted using a Zeiss LSM880 microscope equipped with a 40x 1.3 NA oil objective. Consistency in imaging parameters such as laser power, gain, and offset was maintained across all experiments. Z stacking was performed with 1.0-mm steps in the z-direction, and 512x512-pixel resolution images were analyzed using IMARIS 9.6.2 software (Bitplane) from Bitplane. The IMARIS 'Filaments' function was employed to quantify process length and the number of branch points.

Microglial engulfment was assessed using IMARIS software to generate a 3D surface rendering of the microglia. A threshold was set to ensure accurate reconstruction of microglial processes, which was then utilized for subsequent analyses. VTA-mPFC dopaminergic endings puncta were reconstructed using the IMARIS 'Spots' function. The IMARIS MATLAB-based (MathWorks) plugin 'Split into Surface Objects' was utilized to determine the number of VTA-mPFC dopaminergic endings puncta entirely within the microglial surface. For each group, two images randomly selected from each mouse were reconstructed, each containing at least ten cells, totaling six mice. The mean result was employed for morphological analysis.

**Stereotaxic surgery**

Prior to the precise injection, mice were anesthetized by administering 2% sodium pentobarbital (20 mg/kg) into the abdominal cavity. The mice were then affixed to a stereo locator (Reward, Shenzhen, China) and positioned under a thermal pad to maintain their body temperature. Subsequently, the head hair was trimmed to expose the scalp. The mice's scalps were disinfected with iodine to mitigate the risk of infection during surgery. The scalp was incised, and the skull was exposed and leveled. Following the determination of the target brain area's location with a syringe, it was marked, and the skull was delicately polished with a skull drill to expose the cerebral cortex above the target brain area. A 10-microliter syringe (Gog) equipped with a calibrated glass microelectrode (1B 100-3, WPI, USA) was utilized to administer the virus at 30 nL/min using a microinjection pump (UMP3, WPI, USA). Mice received a 1 µL injection of LPC (1 mg/mL) in the mPFC stereotaxic brain. After surgery, we provided heat pads and additional melon seeds as nutritional support.

In chemogenetic experiments, 200nL of the chemogenetic virus (LV-EF1a-dio-hM4Di-EGFP-WPRE) was injected into the brains of Cx3cr1-CreERT2 mice that had received intrabdominal tamoxifen. The needle was left in place for 10 minutes post-injection to ensure proper diffusion. After 3-4 weeks of complete virus expression, the training stage for the mice was concluded. LPC was injected to interfere with the bilateral mPFC of mice, and subsequently, the test stage commenced. CNO was intraperitoneally injected into mice 30 minutes before each daily test, and the behavioral data were recorded over 5 consecutive days.

For anterograde tracer experiments, 200nL of the virus (AAV-hSyn-DIO-Synaptophysin-mCherry-WPRE-hGH pA 2.45E+12 vg/mL AAV2/9) was injected into the VTA brain region of mice with DAT-Cre. The loss of dopamine endings was observed by introducing LPC into the mPFC brain area.

In validation experiments, AAV-CMV-DIO-mCherry-MIR2885-WPRE-hGH pAA and AAV-U6-CMV-mCherry-TDP43 shRNA virus were used to express miR-2885 and reduce TDP-43. The coordinates for the mPFC brain in mice were set as follows: dorsal to ventral (DV) - 2.00 mm, anterior to posterior (AP) 2.00 mm, lateral (ML) 0.4 mm. For the VTA brain region, coordinates were: DV -4.50 mm, AP -3.50 mm, ML 1.00 mm.

**Optogenetic**

For optogenetic activation of the VTADA-mPFC circuits, the rAAV2/9-Ef1α-DIO-hChR2(H134R) mCherry-WPRE-pA (AAV-DIO-ChR2-mCherry, 4.50E+12 vg/ml, 250nl; BrainVTA) virus was injected into the VTA (250nL) of DAT-Cre mice (n=12 mice/group). The optical fiber with a diameter of 300 lm (FOC-C-W-200-1.25-0.37-4.0; Newdoon Technology Co., Ltd.) was subsequently implanted above the mPFC. The optic fiber ferrule was firmly secured with dental cement for connecting the laser generator.

For optogenetic manipulations, experimenters treated mice 3 days before behavioral testing. On the day of testing, rats were brought to the laboratory and allowed to acclimate to the environment for 4 hours. Rats were anesthetized with isoflurane, and the long-term implanted optical fiber was connected to a laser generator (Shanghai Fiber Laser) through optical cables and sleeves. Rats were then allowed to recover in their home cages for at least 30 min. The Master-8 pulsed stimulator (AMPI) was used to drive a blue-light laser generator (473 nm pulses, 5-8 mW, 15 ms, 20 Hz) to target the stimulated mPFC in the VTA (n = 10). Control rats received the same stimulation (n = 8-10 per group).

**Fiber photometry**

To measure dopamine release, rAAV2/9-hSyn-DA4.4 (AAV-hSyn-DA2m, 5.60E+12 vg/ml, 150 nl) was injected into the mPFC of C57 mice and a recording optical fiber was inserted. mPFC. Diversity. Photometric recordings were performed 7 days after fiber optic implantation using an optical photometric recording system (ThinkerTech) to ensure adequate animal recovery. In the DA release assay, the first six fluorescence signals were recorded, and LPC/saline-induced dopamine release in mice was compared with that of the mice before LPC/saline administration. The start of each event was marked by an optical photometric system activating a timestamp switch (ThinkerTech).

**PLA-douilink (Proximity Ligation Assay)**

The PLA (Duolink; Sigma Aldrich, Cat#DUO92004-100RXN) experiments were conducted on 4% PFA-fixed brain tissue sections. Specific primary antibodies (rabbit anti-PS/mouse anti-C1q) (mouse anti-TDP43/ rabbit anti-p65) were employed to target the proteins of interest. Subsequently, secondary antibodies conjugated to oligonucleotides were introduced into the samples and allowed to incubate. To facilitate ligation, a solution comprising two oligonucleotides and ligase was added. In this particular experiment, oligonucleotides hybridized to two PLA probes, forming a closed loop when brought into close proximity. The amplification phase involved the introduction of an amplification solution, consisting of fluorescently labeled nucleotides and oligonucleotides, along with a polymerase.

The oligonucleotide arms of the PLA probe served as primers, initiating rolling circle amplification with the linker loop as a template, ultimately generating a tandem product. Fluorescently labeled oligonucleotides then hybridized to the resulting RCA product. The visualization of PLA signals was achieved through the observation of transparent fluorescent spots, which were subsequently detected using confocal microscopy (Olympus BX43+DP74).

**Co-IP assay**

Briefly, cell lysates were combined with 1.2 mL of a dilute buffer (lysis buffer) and incubated overnight at 4°C with Protein A/G Agarose (sc-2003; Santa, Dallas, TX, USA) and rabbit anti-P65 antibody (1:1000, ab16502; abcam,). The subsequent day, the agarose beads were harvested through centrifugation at 5000 rpm for 15 minutes at 4°C. After collection, the beads underwent elution two to three times and were subjected to boiling to denature the protein–bead complex. The proteins were then separated using SDS-PAGE, followed by electrophoretic transfer onto 0.22 µm nitrocellulose membranes. Immunoblotting was carried out using primary antibodies, namely mouse anti-TDP-43 antibody (1:1000, SC-376311, Santa, Dallas, TX, USA) and rabbit anti-P65 antibody (1:100, ab16502; abcam).

**Electrophysiology**

The mice were initially anesthetized with isoflurane, followed by intraperitoneal administration of pentobarbital (20mg/kg). Subsequently, 20 mL of oxygenated ice-cold N-methyl-D-glucosamine (NMDG) and artificial cerebrospinal fluid (ACSF) were injected into the left heart of the mice to eliminate blood from the brain. The primary components of NMDG included (unit: mM): 2.5 KCl, 0.5 CaCl2, 1.2 NaH2PO4, 30 NaHCO3, 5 Na-ascorbate, 3 NA-pyruvate, 25 glucose, 20 n-2-hydroxyethyl piperazine-N'-2-ethane sulfonic acid (HEPES), 10 MgSO4, 3 glutathione (GSH), and 2 thiourea (pH: 7.3-7.4, osmotic pressure: 300-310 mOsm/kg). Post-perfusion, brain tissue was placed on a microtome (VT1200s, Leica, Germany) in a frozen (2-4℃) NMDG ACSF and vibrated at a rate of 0.18 mm/s. Acute brain sections (300 mm) containing the medial prefrontal cortex (mPFC) were then cut. The brain sections were initially incubated in pre-oxygenated NMDG solution for 15 minutes and subsequently recovered in ACSF at 25℃ (HEPES) for at least 1 hour. The ACSF composition included (in mM): 92 NaCl, 2.5 KCl, 2.5 KCl, 2 CaCl2, 1.2 NaH2PO4, 30 NaHCO3, 5 Na-ascorbic acid, 3 NA-pyruvate, 25 glucose, 20 HEPES, 2 MgSO4, 3 GSH, and 2 thiourea (pH: 7.3-7.4, osmotic pressure: 300-310 mOsm/kg).

Subsequently, the brain slice was transferred to another slice, with continuous exposure to standard ACSF containing (in mM): 129 NaCl, 3 KCl, 2.4 CaCl2, 20 NaHCO3, 10 glucose, 1.3 MgSO4, and 1.2 KH2PO4 (pH: 7.3-7.4, osmotic pressure: 300-310 mOsm/kg). The standard ACSF temperature was maintained at 32 °C using an in-line solution heater (TC-344B, Warner Instruments) at a flow rate of 2.5-3 mL/min.

To visualize the morphology of neurons, cells were observed through a stereoscopic microscope, and neuronal signals were subsequently collected using a patch clamp and pCLamp software. To record action potentials of vertebral neurons in the prefrontal cortex, action potentials were induced by applying currents of varying intensity. The number of action potentials and the minimum current intensity were recorded, followed by analysis using specialized software. Clampfit software version 10.7 (Axon Instruments, Inc., USA) and MiniAnalysis software version 6.03 (Synaptosoft Inc., USA).

**Real-time PCR (qPCR)**

The pre-treated cells were detached from the medium, rinsed with PBS, and each dish was treated with 1 mL Trizol. The cells were then homogenized, transferred to 1.5 mL EP tubes, and mixed with 200 µL chloroform. After vigorous mixing, the mixture was left to stand for 15 minutes, followed by extraction of the supernatant, which was then mixed with an equal volume of isopropyl alcohol. Subsequent centrifugation led to the removal of the supernatant, and the pellet was washed with anhydrous ethanol several times. The final step involved drying and reconstituting the pellet in ribonuclease-free water, with the nucleic acid concentration assessed using a dedicated machine.

The RNA obtained underwent reverse transcription into cDNA through PCR reverse transcription. RT-qPCR was conducted using an ABI7500 quantitative PCR instrument (7500, Applied Biosystems, Carlsbad, CA, USA) under the following conditions: initial denaturation at 95℃ for 10 minutes, followed by denaturation at 95℃ for 10 seconds, annealing at 60℃ for 20 seconds, and extension at 72℃ for 34 seconds. Each primer pair was set up in triplicate, and the reaction systems included 25 µL VerQuest SYBR Green one-step qRT-PCR Master Mix (2×), 0.5 µL VerQuest 100X RT Enzyme Mix for SYBR Green Assay, 2.5 µL forward primer (10 µM), 2.5 µL reverse primer (10 µM), 1 µL Template RNA, and 50 µL of RNase-free water and diethylpyrocarbonate (DEPC)-treated water. Glyceraldehyde-3-phosphate dehydrogenase (GAPDH) and U6 were employed as internal reference parameters. The primers were synthesized by Shenggong (Beijing, China), and the reliability of the PCR results was verified using the melting curve. The amplification curve threshold (CT value) was calculated using the formula: ΔCt = target gene (CT) − internal reference (CT). The relative quantification method was utilized to calculate the expression of target gene pairs according to 2^−ΔCt. Each experiment was repeated three times to obtain an average value.

**Luciferase reporters assay**

The predicted target gene of miR-2885 was identified through RNA-seq results, and dual luciferase reporter gene assays were employed to validate whether TDP43 served as the direct target of miR-2885. The 3'UTR wild type (wt) and mutant type (mut) of TDP43 were ligated to the pGL3-Basic vector, resulting in two distinct groups: TDP43-WT and TDP43-MUT. BV2 cells were transfected with the plasmids using Lipofectamine 3000 (Cat#L3000008, Thermo Fisher).

Upon reaching 70% cell density, lip3000 reagent was added to serum-free medium, and the plasmids were combined in a separate serum-free medium. Subsequently, the lip3000 mixture was added to the plasmid mixture, incubated for 10-15 minutes, and then introduced to the cells in serum-free medium. Six hours after transfection, the culture medium containing serum was replaced, and cells were harvested 48 hours post-transfection. The cells were lysed, followed by centrifugation for 3-5 minutes, and the supernatant was collected.

Fluorescence intensity was determined using a dual luciferase kit, where each sample (50 μL) was mixed with 100 μL of detection reagent using a luminometer. The cell lysate reporting the gene served as the control. The relative luciferase unit (RLU) was measured with a fluorometer. RL activity was utilized as an internal control, and the relative luciferase activity was calculated by the ratio of RLU of firefly luciferase activity to RLU of RL activity.

**Western blot**

Bilateral medial prefrontal cortex (mPFC) tissue samples were extracted from mice. The PFC samples were homogenized in lysis buffer (Beyotime, P0013B, Shanghai, China) containing a protease/phosphatase inhibitor cocktail (Beyotime, P1050). The homogenate was centrifuged (12,000 × g) at 4°C for 30 minutes, and the resulting supernatant was collected. Protein levels in the brain supernatant were determined using the Micro Bicinchoninic Acid Protein Assay Kit (23235, Thermo Fisher Scientific, Waltham, MA, USA) with bovine serum albumin as the standard. Protein isolation was carried out through sodium dodecyl sulfate-polyacrylamide gel electrophoresis (36250, Yeasen Biotechnology, Shanghai, China), and the proteins were subsequently electrophoretically transferred to nitrocellulose or polyvinylidene fluoride film (10600023, GE Amersham Biosciences, Little Chalfont, Buckinghamshire, UK). The membrane was sealed with blocking buffer (5% skim milk) at room temperature (20-25 °C) for 2 hours. Following this, the membrane was incubated with primary antibodies, including an overnight incubation at 4°C. After washing, the membrane was incubated with secondary antibodies, and an Odyssey laser imaging scanner was used for imaging. Protein content analysis was performed using Image J.

The antibody dilutions used were as follows: rabbit anti-iNOS (1:200, HUABIO), rabbit anti-COX2 (1:200, HUABIO), rabbit anti-TNF-ɑ (1:200, HUABIO), mouse anti-TDP-43 (1:200, HUABIO), rabbit anti-p65 (1:200, HUABIO), mouse anti-STAB-1 (1:200, HUABIO).

**Cell cultures**

The mouse microglial cell line BV2 (ATCC, Manassas, VA) was maintained in DMEM (BasalMedia Technologies, L130KJ) supplemented with 10% FBS and 1% penicillin–streptomycin solution, and incubated at 37°C in a 5% CO2 environment. Lysophosphatidylcholine (LPC) at a concentration of 1 mg/mL was introduced into the culture medium. After 24/48 hours, both mRNA and protein were collected for further analysis.

**Microglia isolation**

Experimental mice were stratified into two groups: Saline group and mPFC group, both injected with LPC in the brain region, comprising 6 mice per group. Following 3 days of LPC and saline injections, the mice were anesthetized with 2% pentobarbital sodium. Subsequently, PBS was administered to extract brain tissue, specifically targeting the mPFC brain region, which was then carefully collected using tweezers and placed in a 1.5 mL enzyme-free EP tube. To initiate the isolation process, 1 mL of 0.25% trypsin was added to the brain tissue, which was then homogenized. After gentle agitation with a 1 mL pipette gun, the mixture was incubated in a 37°C cell incubator for digestion. The resulting digested cell suspension was transferred to a 15 mL centrifuge tube, followed by the addition of 10 mL DPBS buffer. A room temperature centrifugation for 5 minutes was performed, and the supernatant was aspirated. The cells were then suspended in preheated medium. The subsequent day, the medium was replaced to eliminate impurities. For microglia collection, 1 mL of 0.25% trypsin was added to the cells and incubated for 3 minutes. A gentle tap on the T-25 culture bottle allowed the suspended cells to be collected from the medium into a 15 mL centrifuge tube. Following the addition of 10 mL DPBS, centrifugation for 5 minutes occurred, and the supernatant was discarded. Adding 90 μL PB buffer, the suspended cells were gently agitated. Subsequently, 10 μL of microglia CD11b sorting magnetic beads were introduced. The mixture was thoroughly blended at 4°C and incubated for 15 minutes, away from light. The cells were then washed with 1 mL of pre-cooled PB buffer and centrifuged for 5 minutes. After completely removing the supernatant, the cells were suspended in 1500 μL PB buffer. Utilizing a suitable magnetic cell sorter (MACS), a sorting column was positioned in the magnetic field and cleaned with 500 μL PB buffer. The prepared cell suspension was added to the sorting column, and this process was repeated three times. Microglia were acquired by adding 1 mL PB buffer to the sorting column, pipetting, and flushing the magnetically labeled cells.

**Enzyme-linked immunosorbent assay**

Human serum samples were assayed using an enzyme-linked immunosorbent assay (ELISA) with the LPC ELISA Kit [human] (Cat# v03147h, Beyotime, China). The procedure was executed in accordance with the manufacturer's instructions. Optical density values were measured using a microplate reader (BioTek, Hercules, CA, USA).

**Quantification and statistical analysis**

In the multiple-group comparative analysis of clinical data through violin plots, we employed One-way ANOVA analysis of variance alongside the LSD/SNK method. Pearson correlation was utilized to assess the relationship between LPC levels (pg/ml) and scores from the MoCA and MMSE tests. Spearman correlation was applied to examine the correlation between LPC levels (pg/ml) and the clock drawing test results. Diagnostic accuracy was determined by constructing a receiver operating characteristic (ROC) curve to calculate the area under the curve (AUC). Categorical data were expressed as percentages (%) and compared using the Chi-square test. All statistical analyses were conducted using SPSS Statistics (version 19.0, IBM Corp., Armonk, NY, USA).

For experimental data, Fiji software (http://imagej.nih.gov/ij/) performs semi-quantitative analysis of protein and fluorescence intensity. The statistical analysis and graphical representation were performed using GraphPad Prism 8 (GraphPad Software, Inc., USA). Electrophysiological recording data were analyzed offline using Clampfit software version 10.79 (Axon Instruments, Inc., USA) and MiniAnalysis software version 6.03 (Synaptosoft Inc., USA). Microglia morphology was visually rendered in three dimensions using Imaris software. Q-Q plots were employed for hypothesis testing before conducting T-tests, ANOVA, and descriptive statistics.

Statistical comparisons between two groups were executed using the Student t-test. For multi-experimental analysis, One-way analysis of variance (One-way ANOVA) and two-way analysis of variance (Two-way ANOVA) were employed according factors. Data were presented as individual values or mean ± SEM, with significance levels indicated as *P < 0.05, **P < 0.01, ***P < 0.001, ****P < 0.0001.

**References**

Blandini, F. and M. T. Armentero (2012). "Animal models of Parkinson's disease." FEBS J 279(7): 1156-1166.

Mustapha, M. and C. N. Mat Taib (2021). "MPTP-induced mouse model of Parkinson's disease: A promising direction of therapeutic strategies." Bosn J Basic Med Sci 21(4): 422-433.
