# Supplementary material for: Enhanced Microglial Engulfment of Dopaminergic Synapses Induces Parkinson's Disease‐Related Executive Dysfunction in an Acute LPC Infusion Targeting the mPFC
Source: Aging Cell. 2025 Feb 15;24(5):e70003. doi: 10.1111/acel.70003 (PMC12073916; doi:10.1111/acel.70003)
Supplement: Supplementary file 3 — File S3. [file ACEL-24-e70003-s003.docx]

**Key resources table**

| **REAGENT or RESOURCE** | **SOURCE** | **IDENTIFIER** |
| --- | --- | --- |
| **Antibodies** |  |  |
| Rabbit anti-iNOS | Cell signaling | Cat#13120S |
| Rabbit anti-COX2 | Cell signaling | Cat#4842S/12282T |
| Rabbit anti-TNFα | Affinity | Cat#AF7014 |
| Rabbit anti-GDNF | Abcam | Cat#ab176564 |
| Rabbit anti-PS | MyBioSource | Cat#MBS832879 |
| Mouse anti-CD11b | Proteintech | Cat#6519-1-AP |
| Mouse anti-STAB1 | SANTA | Cat#SC-293254 |
| Rabbit anti-p65 | Cell signaling | Cat#8242S |
| Rabbit anti-Iba1 | Huabio | Cat#ET1705-78 |
| Mouse anti-MHC | Abcam | Cat#ab23990 |
| Mouse anti-TDP43 | SANTA | Cat#SC-376311 |
| Mouse anti-CD68 | Abcam | Cat#ab955 |
| Mouse anti-C1q | Abcam | Cat#ab11861 |
| Rabbit anti-Histone H3 | Abcam | Cat#ab1791 |
| IgG H&L （Alexa Fluor 488) | Abcam | Cat#ab150113 |
| IgG H&L （Alexa Fluor 594) | Abcam | Cat#ab150080 |
| IRDye®800CW Goat anti-Rabbit IgG secondary antibody | LI-COR | Cat#926-32211 |
| IRDye®680RD Goat anti-Mouse IgG secondary antibody | LI-COR | Cat#926-68070 |
| **Virus** | | |
| rAAV-hSyn-DIO-Synaptophysin-mCherry-WPRE-hGH pA | BrainVTA | Cat#PT-2755 |
| rAAV-hSyn-DIO-Synaptophysin-EGFP-WPRE-hGH pA | BrainVTA | Cat#PT-4595 |
| LV-EF1a-dio-hm4d-EGFP-WPRE | BrainVTA | Cat#LV-0919 |
| rAAV-CMV-DIO- mCherry-MIR2885-WPRE-hGH pAA | BrainVTA |  |
| LV-MIR2885-up | GENE |  |
| LV-Tardbp-RNAi | GENE |  |
| **REAGENT or RESOURCE** | | |
| LPC | Sigma Aldrich | Cat#L4129 |
| MPTP | Sigma Aldrich | Cat#M0896 |
| Clozapine-N-oxide (CNO) | Sigma-Aldrich | Cat#C0832 |
| Tamoxifen (TAM) | Sigma-Aldrich | Cat#T5648 |
| PSVue550 | Molecular Targeting Technologies | Cat#P1005 |
| SKF 38393 hydrochloride | MCE | Cat#SKF-38393A |
| Lipofectamine 3000 | Thermo Fisher | Cat#L3000008 |
|  |  |  |
| **Critical Commercial Assays** | | |
| Dual Luciferase Reporter Gene Assay Kit | Beyotime | Cat#RG027 |
| TIANprep Midi Plasmid Kit | TIANGEN | Cat#DP106 |
| Human Lysophosphatidylcholine(LPC) ELISA Kit | Beyotime | Cat#v03147h |
| Mouse Lysophosphatidylcholine(LPC) ELISA Kit | CIOBO BIO | Cat#CB11317-Mu |
| miRcute Plus miRNA First-Strand cDNA Kit | TIANGEN | Cat#KR211 |
| miRcute Plus miRNA qPCR Kit | TIANGEN | Cat#FP411 |
| Duolink Kit | Sigma Aldrich | Cat#DUO92004 |
|  |  |  |
| **Experimental models: organisms/strains** | | |
| Mouse: wild type C57BL/6J |  |  |
| Mouse: *DAT-Cre* |  |  |
| Mouse: *Cx3cr1-GFP* | Jackson Laboratory | 005582; RRID: IMSR_JAX:005582 |
| Mouse: *Cx3cr1-Cre^ERT2^* | Jackson Laboratory | 021160; RRID: IMSR_JAX:021160 |
| **Oligonucleotides** | | |
| qPCR: Mouse STAB1 forward | This paper | CACGACACGCTGGCAGATACAC |
| qPCR: Mouse STAB1 reverse | This paper | GGAGGCAACAAGACCTGGCTAAC |
| qPCR: Mouse C3 forward | This paper | GTTCGGCATAGAGAAGAGGCAAGAG |
| qPCR: Mouse C3 reverse | This paper | TTGTTGAAGGCAGCATAGGCAGAG |
| qPCR: Mouse C3R forward | This paper | CCTTCGTGCCATCATCTCAGTGTAG |
| qPCR: Mouse C3R reverse | This paper | GGTGGTGGTGCTCATCTGGTTAC |
| qPCR: Mouse CX3CR1 forward | This paper | TCTGTTGGTGGTCCTCGCTCTC |
| qPCR: Mouse CX3CR1 reverse | This paper | AGGTAGTGAGTCCAGAAGGGCAAG |
| qPCR: Mouse Iba1 forward | This paper | CTGGGAGTTAGCAAGGGAATGAGTG |
| qPCR: Mouse Iba1 reverse | This paper | GTTGTCTTAGGCTGGCAGTCTGTC |
| qPCR: Mouse tmem119 forward | This paper | GGCTGACATTCTGGCTGCTACC |
| qPCR: Mouse tmem119 reverse | This paper | GCACCTCTTCCTCCTCCTCCTC |
| qPCR: Mouse TARDBP forward | This paper | CTGTGCTTCCTCCTTGTGCTTCC |
| qPCR: Mouse TARDBP reverse | This paper | CAACACCGTCCCATCGTCTTCTG |
| qPCR: Mouse miR-2885 forward | This paper | TTAACATATCGGCGGCAAGCGC |
|  |  |  |
| **Software and Algorithms** | | |
| GraphPad Prism 8.0 | GraphPad Software |  |
| IMARIS 9.6.2 | Bitplane |  |
| ImageJ | National Institutes of Health |  |
| Image studio | National Institutes of Health |  |
| Illustrator |  |  |
| Clampfit | Molecular Devices |  |
| LAS X | Leica |  |
|  |  |  |
