# Supplementary material for: Enhanced Microglial Engulfment of Dopaminergic Synapses Induces Parkinson's Disease‐Related Executive Dysfunction in an Acute LPC Infusion Targeting the mPFC
Source: Aging Cell. 2025 Feb 15;24(5):e70003. doi: 10.1111/acel.70003 (PMC12073916; doi:10.1111/acel.70003)
Supplement: Supplementary file 4 — Data S1. [file ACEL-24-e70003-s004.docx]

**Supplementary results**


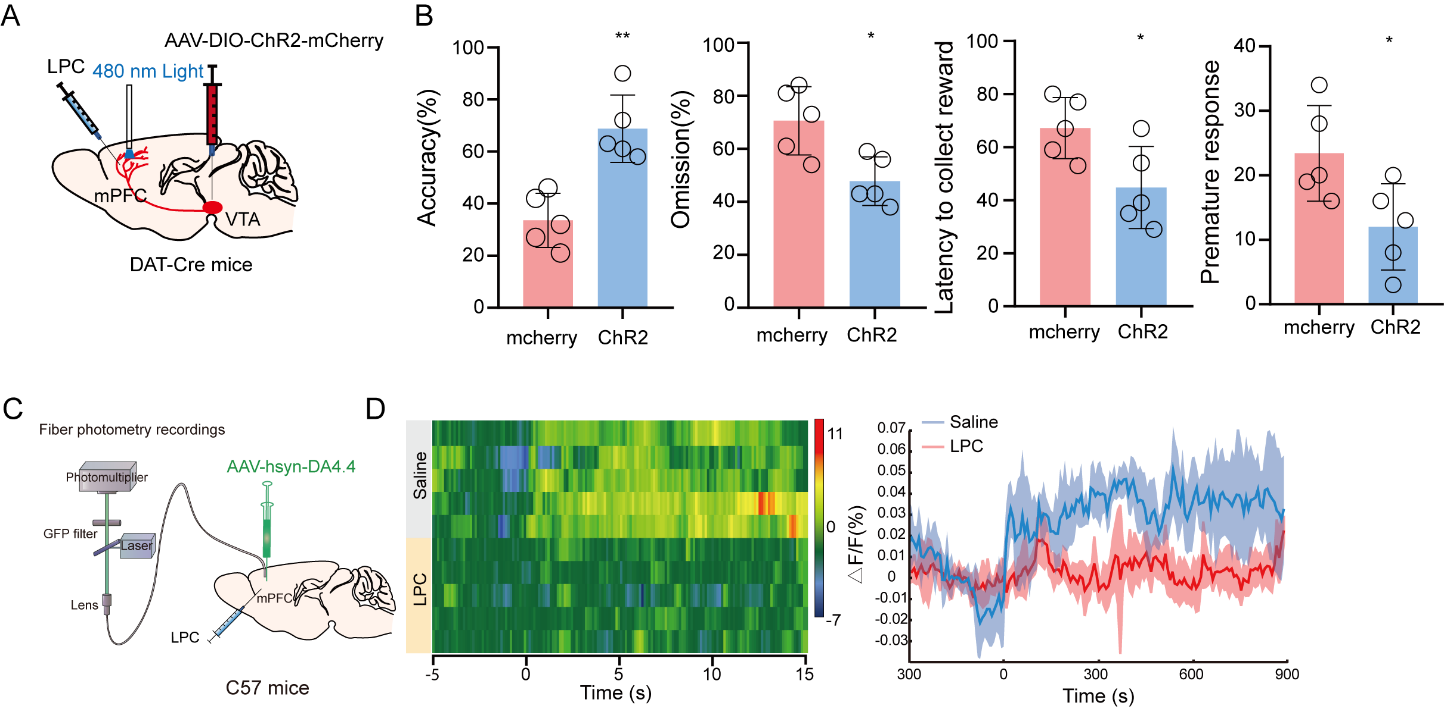


**Fig.S1 Photogenetic activation of the VTA-mPFC circuit improves executive dysfunction in mice induced by LPC, and LPC reduced release of dopamine transmitter in mPFC.**

(A)Schematic for viral injection and optogenetics. (B) Summarized data for accuracy (30% mcherry vs. 70% ChR2) (n=5, t=4.746, P=0.0015), reduced omission (70% mcherry vs. 50%ChR2) (n=5, t=3.231, P=0.0120), decreased latency to collect reward (65 mcherry vs. 40 ChR2) (n=5, t=2.599, P=0.0317), and diminished premature responses (25 mcherry vs. 10 ChR2) (n=5, t=2.558, P=0.0337).(C)Schematic for DA release recording in the mPFC.(D) Averaged responses, heatmaps.


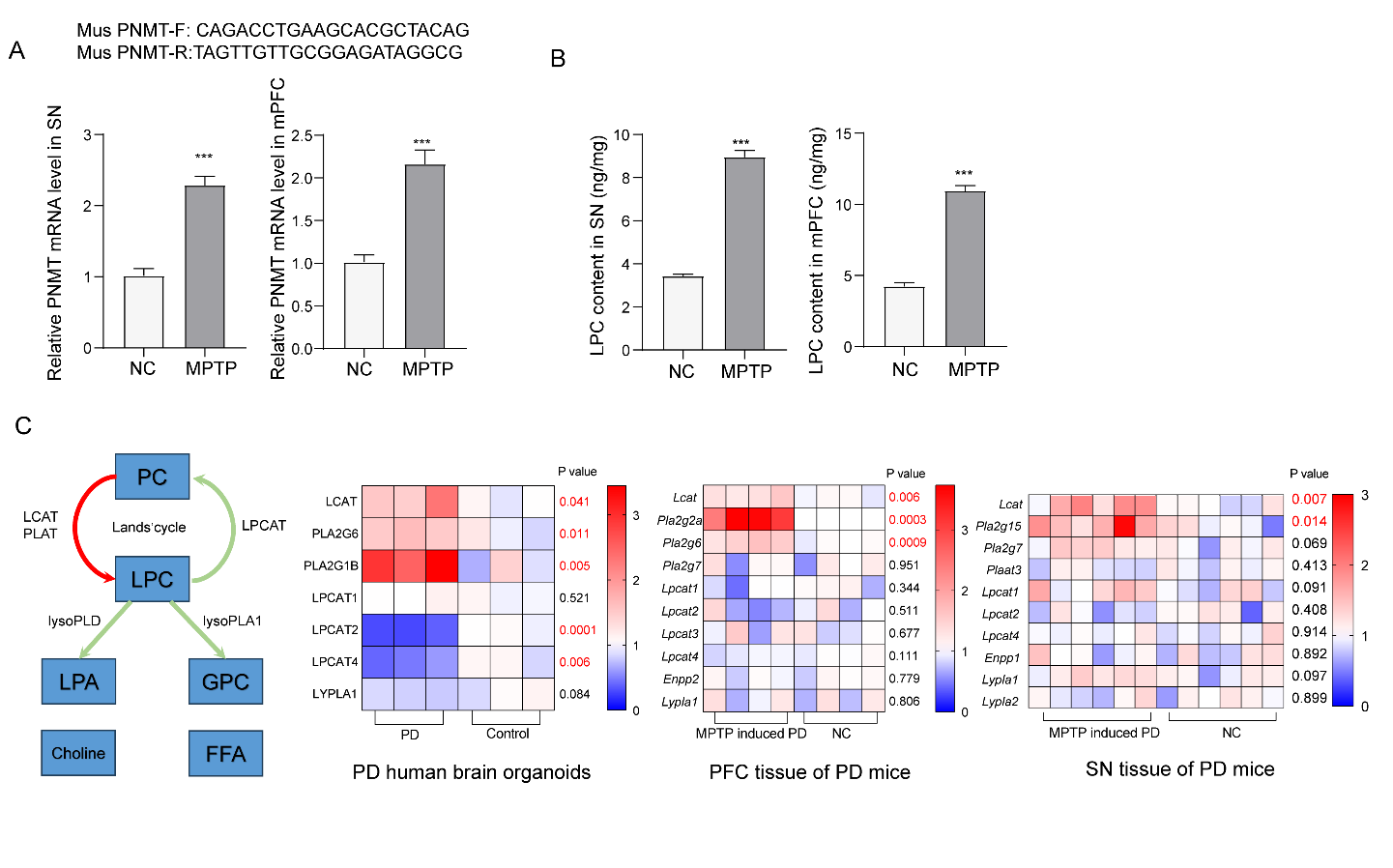


**Fig.S2 PNMT gene expression and LPC content in the prefrontal cortex and substantia nigra, as well as the expression of related genes for the synthesis and breakdown of LPC in human brain tissue, mouse prefrontal cortex, and substantia nigra.**

(A) Quantitative polymerase chain reaction (qPCR) analysis for PNMT gene expression in the prefrontal cortex and substantia nigra. SN(n=4, t=5.934, P<0.0005) mPFC(n=4, t=6.245, P=0.0007) (B) ELISA analysis for LPC content in the prefrontal cortex and substantia nigra. SN(n=5, t=7.293, P=0.0003) mPFC(n=5, t=5.483, P=0.0003 (C) The heatmap illustrates the expression of related genes for the synthesis and breakdown of LPC in human brain tissue, mouse prefrontal cortex and substantia nigra.


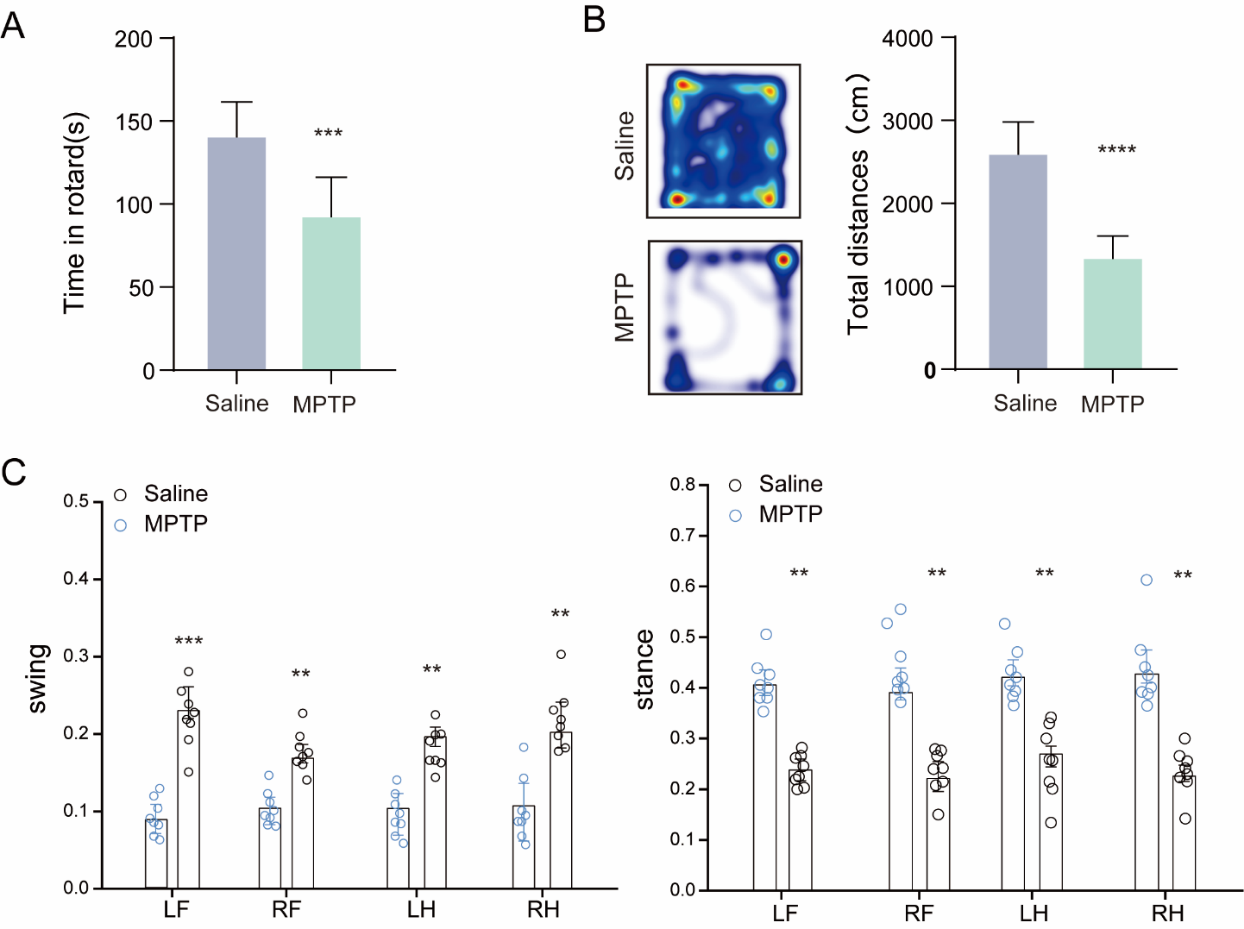


**Fig.S3 MPTP-treated C57BL/6 mice exhibited motor deficits**

(A) Rotard test for Saline group and MPTP group. (n=8, t=6.698, P<0.0001) (B) The open field test for Saline group and MPTP group. (n=8, t=8.232, P<0.0001) (C) Gait analysis for Saline group and MPTP group. (Swing F (1, 18) = 21.56, P=0.0007) (Stance F (1, 18) = 16.28, P=0.0065).


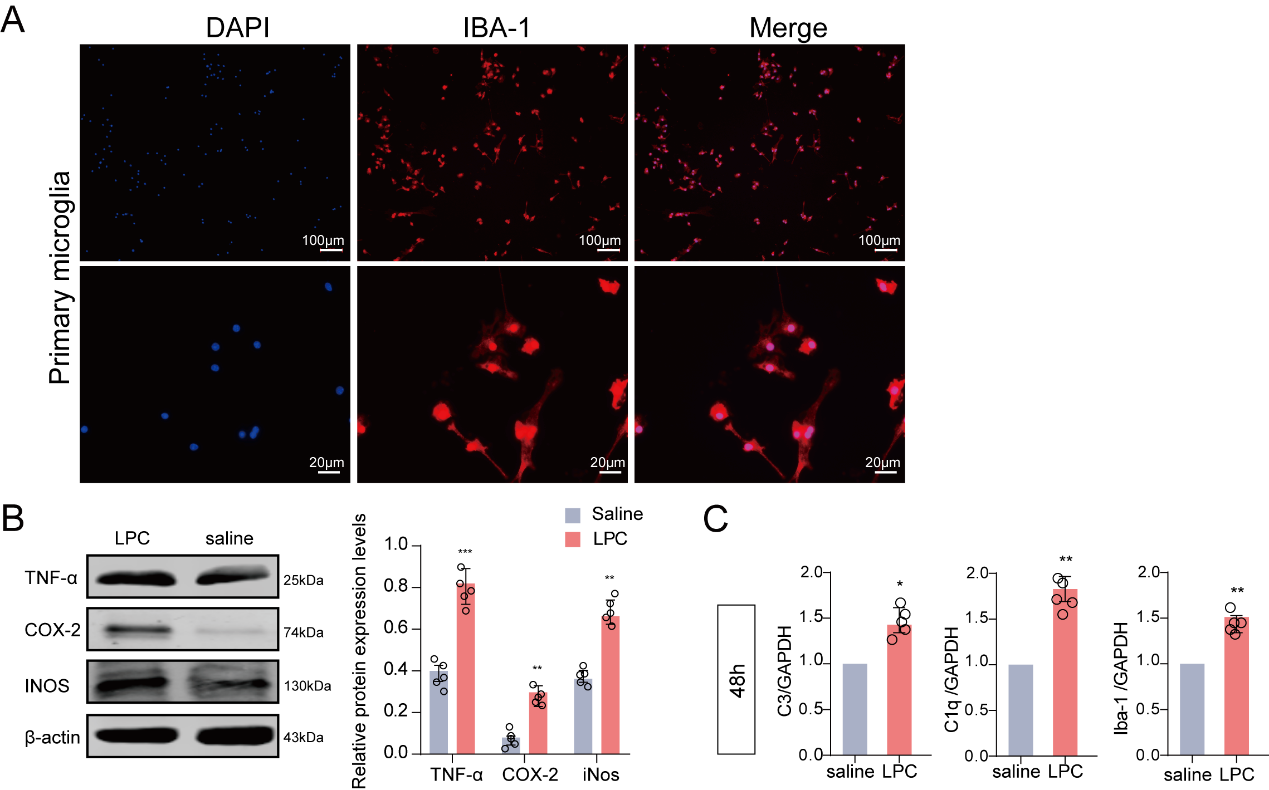


**Fig.S4 The mPFC inflammation induced by LPC in primary microglia**

(A) Immunofluorescence images of primary microglia were extracted. (B) Protein immunoblot bands for the inflammatory cytokines COX-2 and TNF-α, along with Inos in primary microglia. (COX-2, n=5, t=5.283, P=0.0006), TNF-α (n=5, t=4.209, P=0.0038), iNOS (n=5, t=7.283, P=0.0071) (C) qPCR results statistics for C3, C1q, and Iba1 after 48 hours of LPC intervention in primary microglia. C3 (n=5, t=3.187, P=0.0427), C1q (n=5, t=4.273, P=0.0039), Iba1 (n=5, t=7.382, P=0.0046)


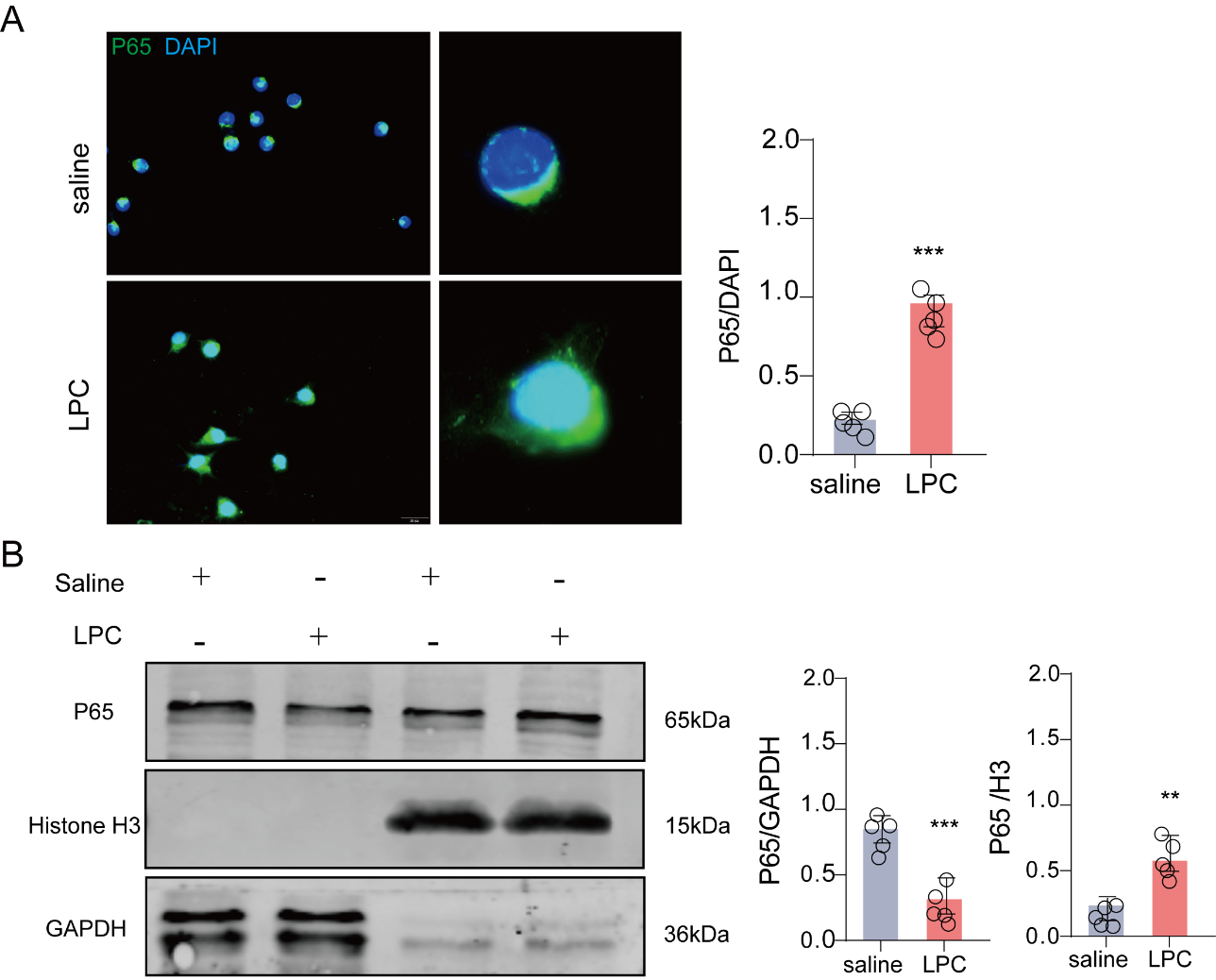


**Fig.S5 LPC intervention resulted in increased nuclear entry of P65 protein**

(A) immunofluorescence staining for the nuclear translocation of P65 after LPC exposure. (n=5, t=7.198, P=0.0006) (B) Western blot experiments for the levels of P65 protein in both the cytoplasm and nucleus following LPC treatment. P65/GAPDH (n=5, t=6.372, P=0.0004), P65/H3(n=5, t=4.632, P=0.0072)


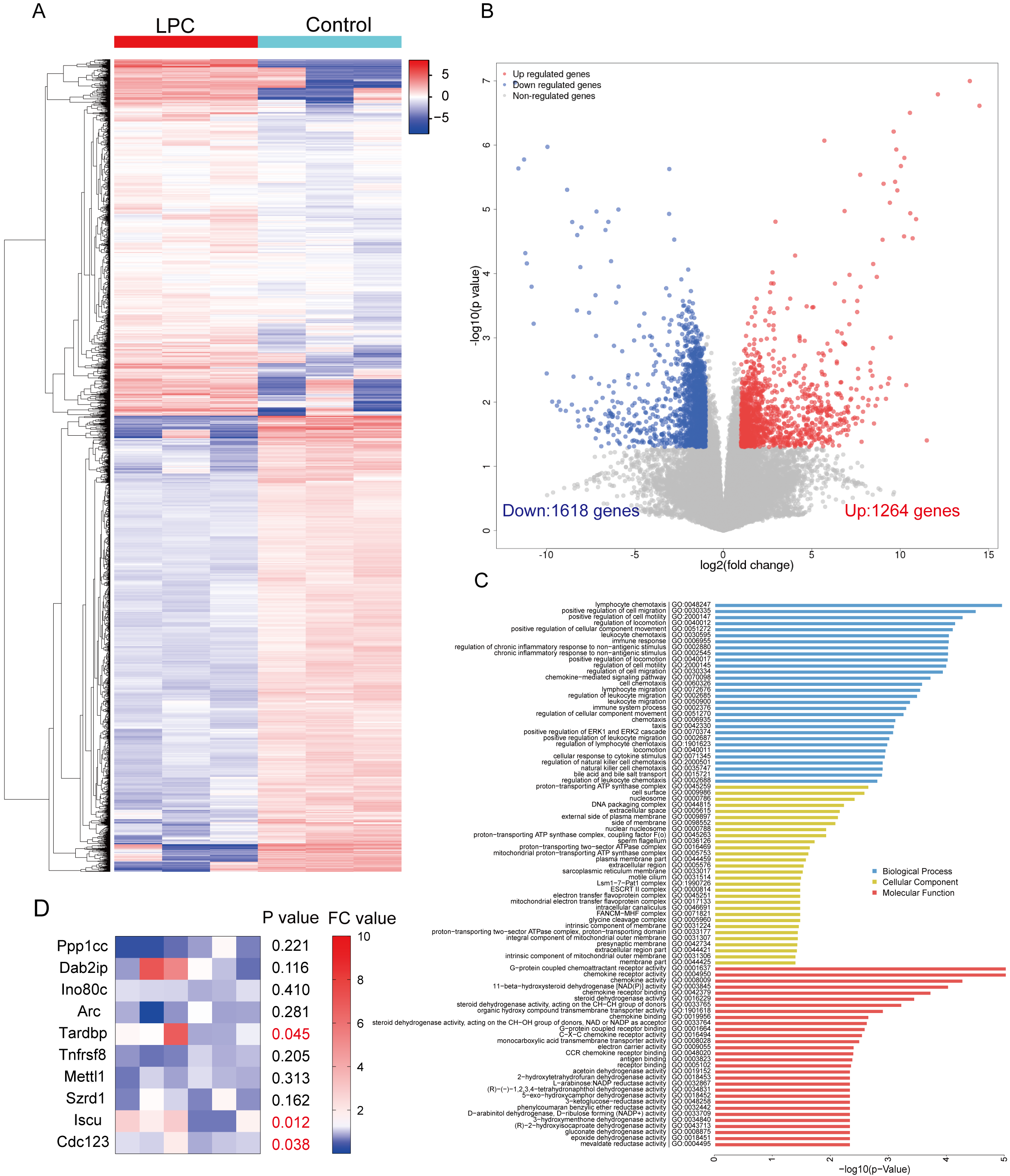


**Fig.S6 Transcriptome sequencing and analysis of differential expression in primary microglia prior to and following LPC stimulation.**

(A) Heat map of differential genes. (B) Volcanic map of differential genes. (C) Go analysis of differential genes. (D) Expression analysis of target genes regulated by miR-2885.


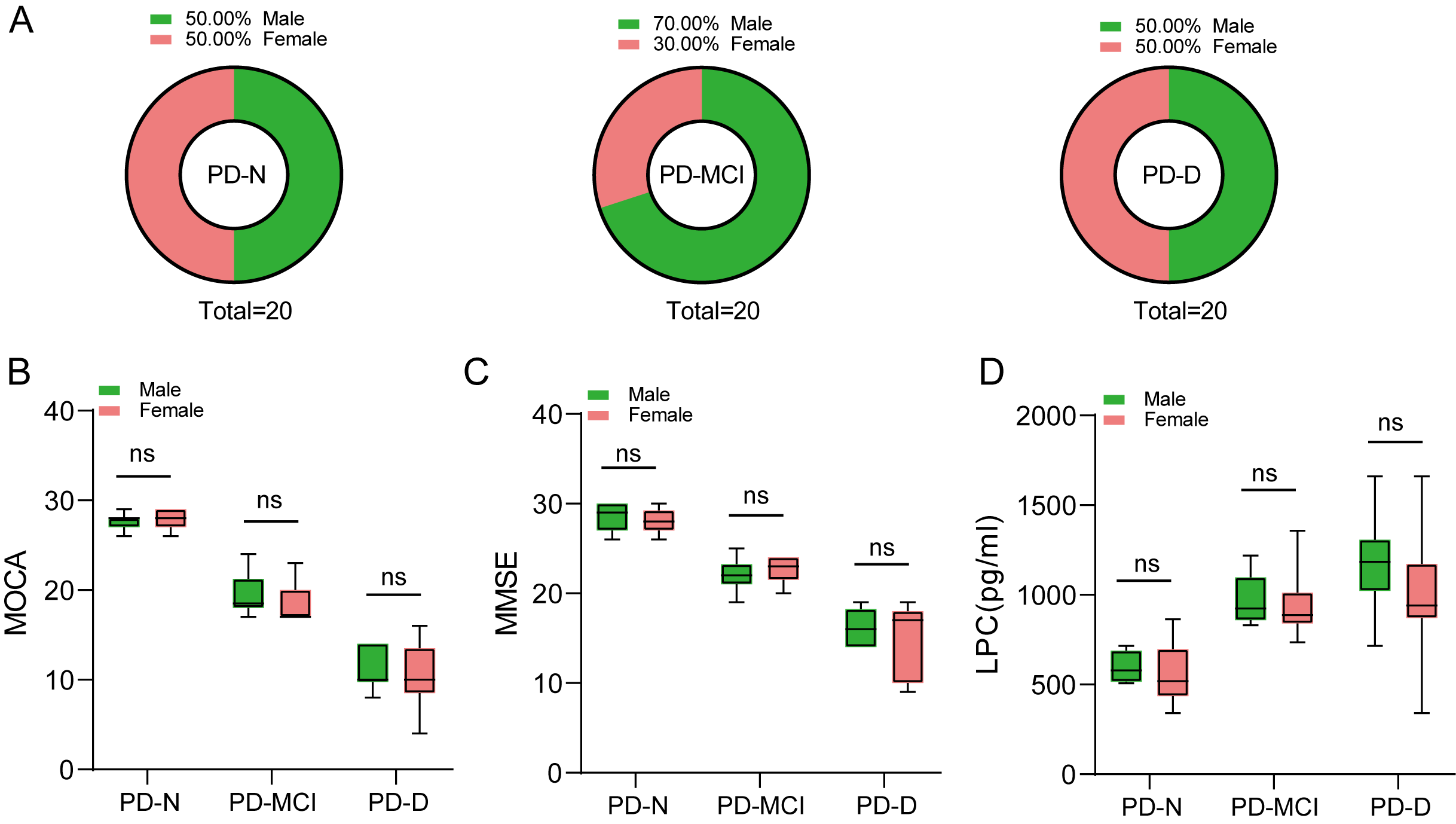


**Fig.S7 Analysis of the cognitive status and LPC content in each subgroup by gender.**

(A) Ratio of male and female gender composition at different cognitive levels within the PD group. (B) A comparison of the cognitive MOCA scores of men and women in each subgroup. (C) A comparison of the cognitive MMSE scores of men and women in each subgroup. (D) A comparison of the LPC serum levels of men and women in each subgroup. ns, non-significant, P>0.05.
